# Pancancer landscape analysis of the thymosin family identified TMSB10 as a potential prognostic biomarker and immunotherapy target in glioma

**DOI:** 10.1186/s12935-022-02698-5

**Published:** 2022-09-26

**Authors:** Ye Xiong, Yanhua Qi, Ziwen Pan, Shaobo Wang, Boyan Li, Bowen Feng, Hao Xue, Rongrong Zhao, Gang Li

**Affiliations:** 1 Department of Neurosurgery, Qilu Hospital, Cheeloo College of Medicine, Institute of Brain and Brain-Inspired Science, Shandong University, Jinan, 250012 Shandong China; 2 Shandong Key Laboratory of Brain Function Remodeling, Jinan, 250012 Shandong China; 3 Department of Neurosurgery, The Frist Affiliated Hospital of Wenzhou Medical University, Wenzhou City, 325000 Zhejiang Province China

**Keywords:** Pancancer, TMSB10, Glioma, Tumor microenvironment, Immune checkpoint blockade

## Abstract

**Background:**

Thymosin family genes (TMSs), biologically important peptides with diverse intracellular and extracellular functions, have been shown to promote the progression of multiple cancers. However, multiomics characterization of TMSs and their role in human cancer prognosis has not been systematically performed.

**Methods:**

We performed a comprehensive analysis of TMSs and thymosin β10 (TMSB10) using multiomics data from more than 10,000 tumor samples of 33 cancer types from The Cancer Genome Atlas (TCGA). We used single-sample gene set enrichment analysis (ssGSEA) and the gene set variation analysis (GSVA) algorithm to investigate the differences in tumor microenvironment (TME) cell infiltration and functional annotation for individual tumor samples, respectively. The role of TMSB10 in the malignant progression of glioma, the promotion of macrophage infiltration,and immunosuppressive polarization, and the combination drug efficacy were assessed via biological function assays.

**Results:**

We comprehensively assessed genomic mutations, expression dysregulation, prognosis and immunotherapeutic response across 33 human cancer samples and showed that TMSB10 is specifically overexpressed in almost all types of cancer tissues. Further pan-cancer analysis showed that TMSB10 is closely related to the biological function, immune regulation and prognosis of glioma. Similar results were also found in several public glioma cohorts and our Qilu local cohort. Further integration with other biological experiments revealed the key roles of TMSB10 in the malignant progression of glioma, the promotion of macrophage infiltration and immunosuppressive polarization. We also identified multiple drugs targeting cells with high TMSB10 expression and validated that knockdown of TMSB10 improved the efficacy of selumetinib (a MEK1/2 inhibitor approved by the FDA for the treatment of neurofibromatosis-associated tumors) and anti-PD1 treatment in glioma.

**Conclusion:**

These results indicate that TMSB10 holds promise as a novel prognostic marker and therapeutic target, providing a theoretical basis for the development of more effective and targeted clinical treatment strategies for glioma patients.

**Supplementary Information:**

The online version contains supplementary material available at 10.1186/s12935-022-02698-5.

## Background

Thymosin family genes (TMSs), including β-thymosin genes (TMSB10, TMSB4X/Y, TMSB15A/B), prothymosin alpha (PTMA) and parathymosin (PTMS), are a series of highly conserved small polypeptides ranging in size from 41 to 45 amino acids that were originally identified in the thymus. It was first reported to prevent actin polymerization as an actin monomer binding protein [1, 2]. Recent studies proved that the thymosin family plays a key role in tumorigenesis and progression [3–10]. Thus, TMSs are key modulators of a variety of human cancers, and systematic studies of TMSs in cancer are necessary and valuable to better understand their role in cancer development and their clinical therapeutic potential.

Immunotherapy, represented by immune checkpoint inhibitors (ICIs), has become the standard of care for a wide range of tumors, but only a small percentage of patients benefit from immunotherapy. Therefore, the screening of new biomarkers that can predict the response to immunotherapy and the development of new immunotherapy combination treatment strategies are crucial for cancer treatment. The tumor microenvironment (TME) significantly influences tumor prognosis, survival outcome, and drug sensitivity. Thymosin was originally discovered in the thymus, an important lymphatic organ of the body, and its function is closely related to immunity [11]. Moreover, TMSB10 induces the immunosuppressive phenotype conversion of tumor-associated macrophages (TAMs) in lung adenocarcinoma [12]. However, there is still no systematic evaluation of TMSs in human cancers in the contexts of microenvironmental immune regulation and cancer immunotherapeutic response.

In this study, we comprehensively investigated the basal expression levels of TMSs in human normal tissues, as well as their genomic mutation, copy number alteration (CNA), expression dysregulation, prognostic implications and immunotherapeutic response implications across 33 types of human cancer samples. The results showed that TMSB10 was specifically overexpressed in almost all types of cancer tissues, and we further comprehensively evaluated the expression pattern, biological function and immunological role of TMSB10 across cancers. TMSB10 was closely related to the biological function, immune regulation and prognosis of glioma. Similar results were found in multiple other publicly available glioma datasets and our local Qilu dataset. We also characterized the multiomics molecular features of TMSB10 in glioma, identifying various novel dysregulated pathways. Further integration with other biological experiments revealed the key roles of TMSB10 in glioma proliferation, invasion, and mesenchymal transformation. Moreover, high TMSB10 expression was associated with high macrophage infiltration and immunotherapy resistance in gliomas. We also identified multiple drugs targeting high TMSB10 expression and validated that knockdown of TMSB10 improved the efficacy of selumetinib (a MEK1/2 inhibitor approved by the FDA for the treatment of neurofibromatosis-associated tumors) and anti-PD1 treatment in glioma. These results indicated that TMSB10 holds promise as a novel prognostic marker and therapeutic target, providing a theoretical basis for the development of more effective and targeted clinical treatment strategies for glioma patients.

## Methods

### Qilu cohort patients and specimens

Human glioma tissues and normal brain tissues (the cortex of decompressive surgery patients with brain trauma or hypertensive intracerebral hemorrhage) were obtained from patients admitted to Qilu Hospital from November 2017 to December 2019. All participants provided written informed consent, and the research was approved by the Ethical Committee on Scientific Research of Shandong University Qilu Hospital (Approval number: KYLL-2018-324). The RNA-seq data of our local glioma samples have been deposited in the Genome Sequence Archive (GSA) under Accession number CRA002339, and data were released when the paper was published. The processed data are available from the corresponding author upon reasonable request.

### Estimation of TME immune cell infiltration

We used the ssGSEA algorithm to quantify the relative abundances of 25 infiltrating immune cells in the GBM TME. Gene sets for tumor-associated BMDMs and tumor-associated MGs were obtained from Bowman et al. [13], and those for 23 other infiltrating immune cell types were obtained from Charoentong et al. [14, 15]. The ESTIMATE algorithm was used to assess the immune and stromal scores and tumor purity of each GBM sample. The proportion of CAFs was estimated using the microenvironment cell population counter (MCP-counter) method, the EPIC tool and the xCell algorithm and transcriptomic fata for heterogeneous tissues.

### Cell lines and cell culture

We purchased the glioma cell lines U87MG and U251 and the human monocyte cell line THP-1 from the Chinese Academy of Sciences Cell Bank. U87MG and U251 cells were cultured in DMEM (Thermo Fisher Scientific, USA) containing 10% FBS (Gibco, USA), 100 U/ml penicillin and 100 mg/ml streptomycin. THP-1 cells were cultured in RPMI-1640 (Thermo Fisher Scientific) supplemented with 10% FBS and were induced to differentiate into macrophages with 100 ng/ml phorbol 12-myristate-13-acetate (PMA) (Sigma–Aldrich; St. Louis, MO, USA). Mesenchymal (MES) subtype GSC cell lines (GSC 20 and GSC 267) were kindly provided by Dr. Frederick F. Lang and Dr. Krishna P.L. Bhat (The University of Texas, M.D. Anderson Cancer Center, Houston, TX, USA) and cultured in DMEM/F12 supplemented with B27 (Invitrogen, USA), 20 ng/ml EGF (R&D Systems, USA), and 20 ng/ml bFGF (R&D Systems, USA). Cells were incubated at 37 °C in a humidified atmosphere of 5% CO2.

### Small interfering RNA and virus transfection

The TMSB10 siRNA, TMSB10 overexpression plasmid and corresponding negative controls were purchased from GenePharma (Shanghai, China). The sequences of TMSB10 knockdown (shRNA) and the corresponding scramble control (shNC) were cloned into the GV112 lentiviral vector to construct lentiviruses (GeneChem, China). A Lipofectamine 3000 kit (Invitrogen) was used for the transfection of siRNAs and plasmids according to the instructions. Sequences are available in Additional file 2: Table S9.

### Quantitative reverse-transcription PCR (qRT–PCR)

Quantitative PCR was performed using SYBR Green PCR Master Mix. mRNA level was normalized by GAPDH. Primers are shown in Additional file 2: Table S9.

### GBO model

GBO models were generated as previously described [16]. GBO medium containing 50% DMEM/F12 (Thermo Fisher Scientific), 50% Neurobasal Medium (Thermo Fisher Scientific, USA), GlutaMax (Thermo Fisher Scientific, USA), nonessential amino acids (NEAAs) (Thermo Fisher Scientific, USA), PenStrep (Thermo Fisher Scientific, USA), N2 supplement (Thermo Fisher Scientific, USA), B27 w/o vitamin A supplement (Thermo Fisher Scientific, USA), 2-mercaptoethanol (Thermo Fisher Scientific, USA), and 2.5 mg/ml human insulin (Sigma, USA) was added to each well, and the plates were was placed on an orbital shaker rotating at 120 rpm at a 37 °C, 5% CO_2_, and 90% humidity in a sterile incubator.

GBOs were cocultured with conditioned medium from GSC 267 cells, transfected with sh-NC or sh-TMSB10, PD1 antibody (5 µM) and/or selumetinib (7.5 µM) for 5 days as indicated. IF staining for Ki67 and CD44 in GBO sections.

### Intracranial mouse mode

Luciferase-labeled U87MG cells (3 × 10^5^ cells per mouse) with TMSB10 knockdown or vector control cells were implanted into the brains of 4-week-old nude mice (Shanghai Laboratory Animal Center (SLAC), Shanghai, China). The growth of intracranial tumors was assessed using bioluminescence imaging (BLI) on the 7th and 10th days. Luciferase-labeled GSC267 cells (5 × 10^5^ cells per mouse) with TMSB10 knockdown or vector control cells were mixed with THP-1 cells (1 × 105 cells per mouse) and injected into the brains of nude mice. Selumetinib was injected intraperitoneally every three days (50 mg/ml) [17]. Bioluminescence imaging was used to measure the tumor volume after 14 days.

### Statistical analysis

Statistical analyses were performed using GraphPad Software 8 (GraphPad Software Inc., CA, USA). For comparisons between two groups, student’s *t* test and Mann-Whitney U test was used for parametric and nonparametric data. For comparisons among more than two groups, the Kruskal–Wallis test and one-way ANOVA were used for nonparametric and parametric data. The cutoff values of each dataset were evaluated based on the association between survival time and TMSB10 using the “survminer” package. The Kaplan–Meier method was used to generate survival curves for the subgroups in each dataset, and the log-rank (Mantel–Cox) test was used to determine if they were significantly different. The HRs for the univariate analyses were calculated using a univariate Cox proportional hazards regression model. Univariate prognostic analysis results were visualized using the “forestplot” R package. Correlations between variables were explored using Pearson or Spearman coefficients. The specificity and sensitivity of TMSB10 in predicting the response to anti-PD1 therapy were assessed by ROC curves, and the area under the curve (AUC) was quantified using the “pROC” R package. P > 0.05 was considered to indicate nonsignificance (ns), and P < 0.05 was considered to indicate statistical significance (*P < 0.05; **P < 0.01; ***P < 0.001, ****P < 0.0001). All data processing steps with R packages was performed using R Studio (version 3.6.3).

## Results

### Pancancer genomic alteration landscape, expression pattern, prognostic significance, and immunological correlation of the thymosin family

**Fig. 1:**
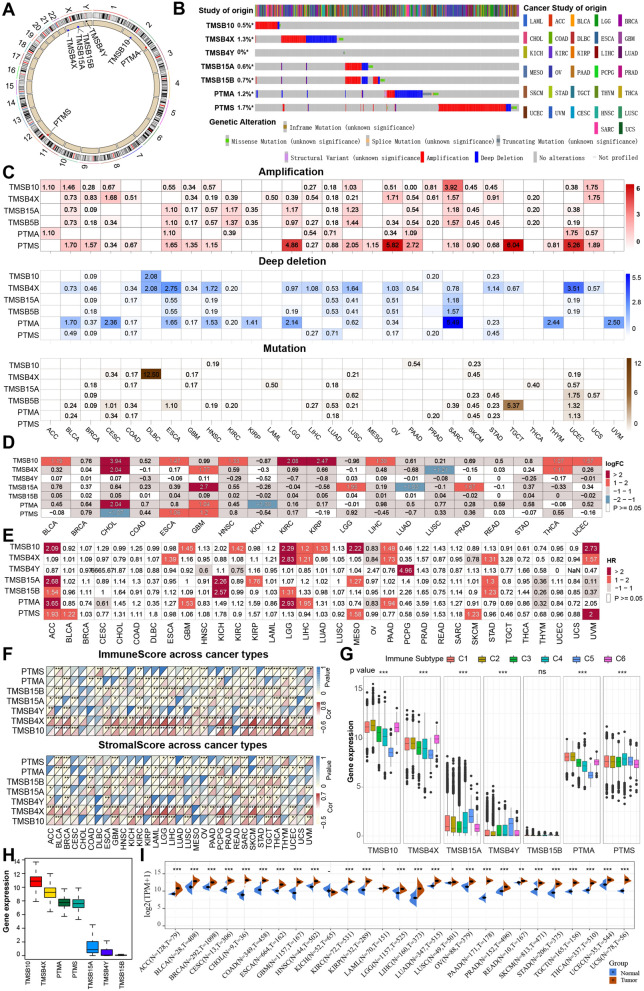
Pancancer genomic alteration landscape, expression pattern, prognostic significance, and immunological correlation of the thymosin family. **A** The location of TMSs on 23 chromosomes. **B** Landscape of genomic aberrations in the TMSs in cancer. Each row represents a gene, and each column represents a patient. Only patients with genomic alterations in the indicated genes are shown. Alteration rates per TMS gene are displayed on the left. **C** Distribution of (up) mutation and (middle and down) CNA frequencies over cancer types. The darkness of color is proportional to the frequency. **D** Differential expression of TMS genes in 19 different cancer types. FC and p values were obtained by comparing normal tissue with the corresponding tumor tissue. Color is displayed only when P value < 0.05. Red indicates upregulation, while blue indicates downregulation. **E** Summary of Cox regression correlation of TMSs with survival. Color is displayed only when P value < 0.05. Red indicates worse survival. **F** Correlation between TMSB10 expression and (up) immune score, as well as (down) stromal score, across 33 cancer types. **G** Differences in the TMSs between six different immune molecular subtypes. The Kruskal–Wallis test was used to determine the significance of differences between the six immune molecular subtypes. **H** Box plot showing the distribution of sample-specific pathway scores across 33 cancer types. The median, interquartile range, and outliers are indicated. **I** The expression of TMSB10 between GTEx normal tissues and tumor tissues. The statistical significance is indicated as follows: ns > 0.05; *P < 0.05; **P < 0.01; ***P < 0.001

To investigate the genomic characteristics of the TMSs in cancer, we first showed the location of thymosin genes on chromosomes, and four of them were located on the sex chromosomes (Fig. 1A). We then calculated the somatic mutation (truncating and missense) and CNA (including amplification and deep deletion) frequencies in a pancancer cohort of 10,953 patients with 32 cancer types. The overall DNA alteration frequency of thymosin family genes was less than 2%, which is relatively low for cancers. PTMS had the highest mutation frequency (1.7%), while TMSB4Y a very low mutation frequency. Moreover, TMSB15A and TMSB15A showed a trend of coamplification or codeletion (Fig. 1B). At the genomic level, CNA, rather than gene mutation, is the primary cause of dysregulation of the thymosin gene in cancer. Subsequently, we showed the distribution of the amplification, deep deletion and mutation frequencies in different cancers. We found that among cancers, several cancer types [such as adrenocortical carcinoma (ACC), mesothelioma (MESO), acute myeloid leukemia (LAML), thyoma (THYM) and uveal melanoma (UVM)] have relatively low mutation frequencies for these genes (Fig. 1C). To assess the expression patterns of these 7 TMSs in cancer tissues, we obtained sample data for 19 cancer types with at least 3 matching tumor and normal samples from the TCGA database and found that these 7 TMSs showed heterogeneous expression patterns across cancer types (Fig. 1D). To further explore the association of thymosin genes with survival, we performed Cox regression analysis for all 7 individual thymosin genes and showed the distribution of hazard ratios (HRs) across 33 cancer types via a heatmap. As shown in Fig. 1E, TMSB10 had significant prognostic value in the largest number of cancer types, acting as an adverse prognostic factor in nine cancer types.

To explore the roles in the immune system and tumor microenvironment, we also investigated the relationship between TMSs and the immune/stromal score, calculated via an estimation algorithm. The expression of TMSB10 and TMSB4X was positively correlated with the immune/stromal score in most cancer types (Fig. 1F). Differences in the expression of thymosin genes between different immune subtypes were further investigated [18]. Expression was significantly different between the C1 (wound healing), C2 (IFN-γ dominant, inflammatory), C3 (lymphocyte depleted), C4 (lymphocyte depletion), C5 (immunologically quiet) and C6 (TGF-β dominant) subtypes, which are characterized by differences in macrophage or lymphocyte signatures. As shown in Fig. 1G, TMSB10 and TMSB4X expression were higher in patients with the C2 and C6 subtypes, which indicate a poorer prognosis, suggesting that TMSB10 and TMSB4X might be involved in tumor immune deregulation.

Analysis of expression data from the Cancer Cell Line Encyclopedia (CCLE) and Genotype-Tissue Expression (GTEx) databases revealed that TMSs were also expressed in various cancer cells (Additional file 1: Fig. S1 A–G) and normal tissues (Additional file 1: Fig. S2 A–G). Furthermore, we found that the expression of TMSB10 in the tumor tissues was the highest among these seven TMSs, and TMSB15A, TMSB4Y and TMSB15B showed relatively low expression (Fig. 1H). In paired pancancer samples for 33 cancer types, TMSB10 expression was found to be significantly higher in tumor tissues than in their corresponding normal tissues in all cancers except for KICH (Fig. 1I), while other genes showed heterogeneous expression patterns (Additional file 1: Fig. S3 A–F), suggesting that TMSB10 might play vital roles in regulating cancer hallmarks and the immune response in cancers. Notably, the expression and function of TMSB10 across cancers, including its involvement in immune-related processes, remain largely unknown. Therefore, we selected TMSB10 as a candidate gene for further study.

### Pancancer expression pattern and biological and immunomodulatory function of TMSB10

**Fig. 2:**
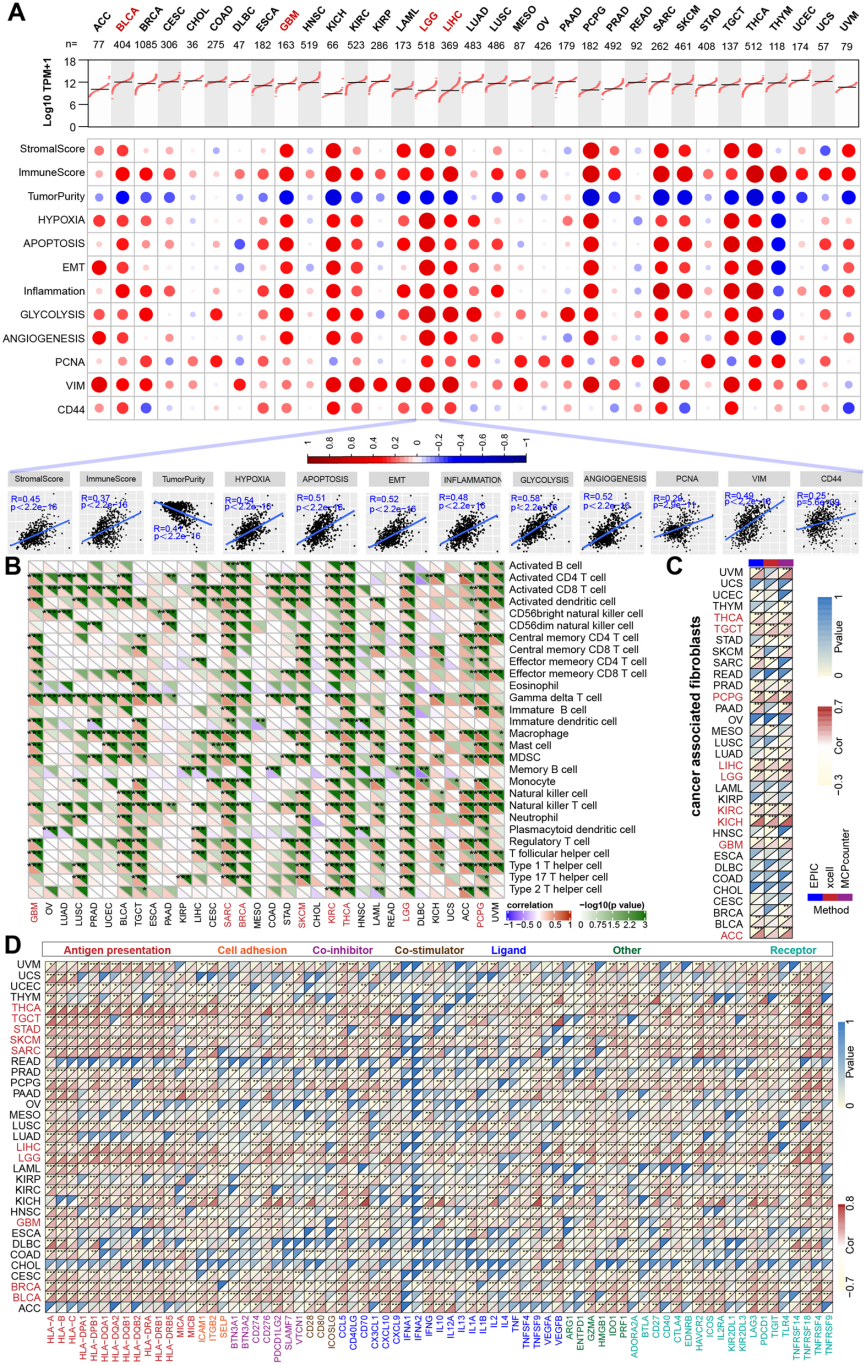
Pancancer expression pattern and biological and immunomodulatory function of TMSB10.** A** (Top) The distribution of sample-specific TMSB10 expression across each cancer type; (middle) bubble plots showing the correlation between TMSB10 and classical cancer pathways or genes. The color of the circle represents the correlation coefficient: red indicates a positive correlation, and blue indicates a negative correlation. (Down) The scatter plot shows the correlation between TMSB10 and classical cancer pathways or genes in LGG. Correlation between TMSB10 and **B** 28 tumor-associated immune cells calculated with the ssGSEA algorithm and correlation with (**C**) CAFs and **D** immunomodulators (immunoinhibitory, immunostimulatory and MHC molecules). The color indicates the correlation coefficient. The asterisks indicate a statistically significant p value calculated using Spearman correlation analysis. The statistical significance is indicated as follows: *P < 0.05; **P < 0.01; ***P < 0.001; ****P < 0.0001

We found that TMSB10 was positively correlated with the enrichment scores of typical cancer hallmarks, the inflammatory response and tumor malignancy markers (PCNA, a proliferation index, and VIM and CD44, markers of invasion) in a wide range of cancer types, especially in lower grade glioma (LGG), glioblastoma (GBM), bladder cancer (BLCA) and liver hepatocellular carcinoma (LIHC) (Fig. 2A). Furthermore, since our results showed that TMSB10 was associated with immunophenotype and inflammatory responses in various tumors, we also focused on its role in immune regulation pathways. Interestingly, we found that a large number of immune-related pathways were positively correlated with TMSB10 in a variety of cancers, such as GBM, LGG, sarcoma (SARC), breast invasive carcinoma (BRCA), skin cutaneous melanoma (SKCM), kidney renal clear cell carcinoma (KIRC), thyroid carcinoma (THCA) and pheochromocytoma and paraganglioma (PCPG) (Fig. 2B). In addition, we observed a statistically positive correlation between TMSB10 expression and the enrichment score of cancer-associated fibroblasts (CAFs), calculated using the EPIC, xCell and MCP-counter algorithms (Fig. 2C), and immunomodulatory molecule analysis (Fig. 2D) in LGG, GBM, LIHC, testicular germ cell tumor (TGCT) and THCA. Cancer-specific univariate prognostic analysis also revealed a significant correlation between TMSB10 and overall survival in multiple cancer types, including ACC, GBM, KIRC, LGG, LIHC, lung adenocarcinoma (LUAD), MESO, pancreatic adenocarcinoma (PAAD) and UVM (Fig. 1E). After a systematic analysis of the relationship between TMSB10 expression, prognosis, biological function and immune regulation, we found a significant relationship between TMSB10 and these variables in gliomas (both in LGG and GBM), suggesting that TMSB10 may be a suitable candidate therapeutic target in glioma.

### Expression signature and prognosis value of TMSB10 in glioma

**Fig. 3:**
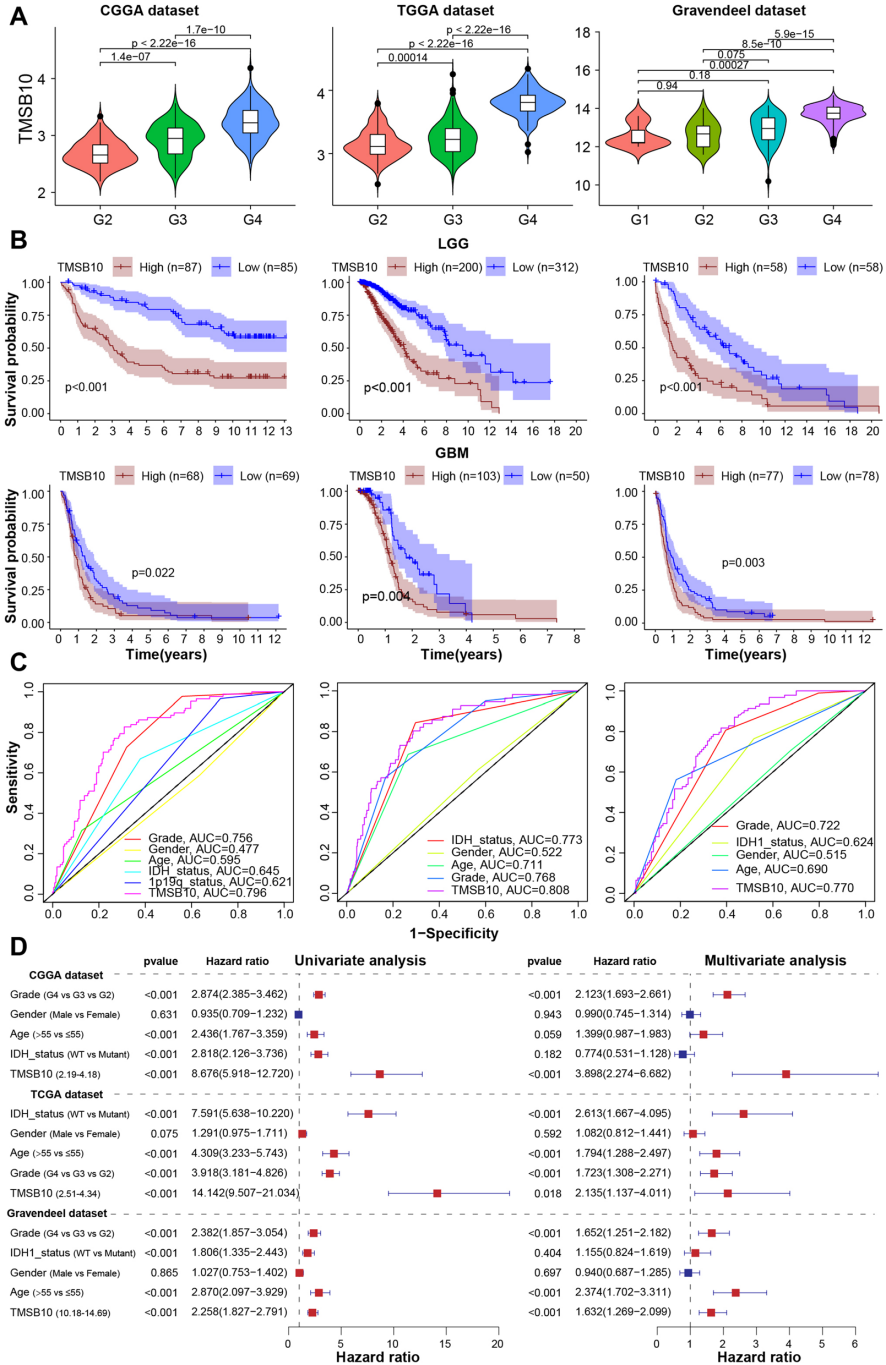
Expression signature and prognostic value of TMSB10 in glioma.** A** The mRNA expression level of TMSB10 increased with tumor grade in the (left) CGGA, (middle) TCGA and (right) Gravendeel datasets. **B** Kaplan–Meier curves for the OS of (upper) LGG and (lower) GBM patients with high TMSB10 expression and low TMSB10 expression in three glioma datasets; the log-rank test was used to calculate the p value. **C** ROC curve showing that, among other clinical characteristics, the expression level of TMSB10 had the largest AUC in predicting the survival rate of glioma patients in three glioma datasets. **D** Univariate and multivariate analyses of TMSB10 expression and clinicopathological characteristics in three glioma datasets

Considering the heterogeneity of different grades of gliomas, we compared the expression levels of TMSB10 in different grades of gliomas in the Chinese Glioma Genome Atlas (CGGA), TCGA and Gravendeel datasets and found that TMSB10 expression was positively correlated with tumor grade, and the expression level was highest in GBM (WHO grade IV glioma) (Fig. 3A). Kaplan–Meier survival curve analysis showed that the prognosis of LGG and GBM patients with high WEE2-AS1 expression was significantly poorer than that of those with low expression in all three glioma datasets (Fig. 3B). Since biological heterogeneity exists between GBM and LGG, we further investigated the prognostic value of TMSB10 in three other GBM datasets. Similarly, we found that the prognosis of patients with high TMSB10 mRNA and protein expression was significantly worse than that of patients with low expression (Additional file 1: Fig. S4 A). Moreover, receiver operating characteristic (ROC) curve analysis of the CGGA, TCGA and Gravendeel datasets showed that TMSB10 expression level could predict glioma survival with AUC values of 0.796, 0.808 and 0.770, respectively, and the survival prediction performance was the better than that of other clinicopathological factors in all three datasets (Fig. 3C). Considering that the prognosis of glioma patients is influenced by various factors, we performed univariate Cox regression analysis to further evaluate the prognostic value of TMSB10 in glioma patients in the CGGA, TCGA and Gravendeel datasets. As shown in Fig. 3D, TMSB10 expression, glioma grade, age, and IDH mutation status were significantly associated with OS. Consequently, we performed multivariate Cox regression analysis and found that the expression level of TMSB10 was an independent prognostic factor for glioma patients in the CGGA, TCGA and Gravendeel cohorts (Fig. 3D). These results suggested that TMSB10 may be a novel prognostic biomarker for glioma.

### Biological pathways and immunological characteristics of TMSB10 in glioma

**Fig. 4:**
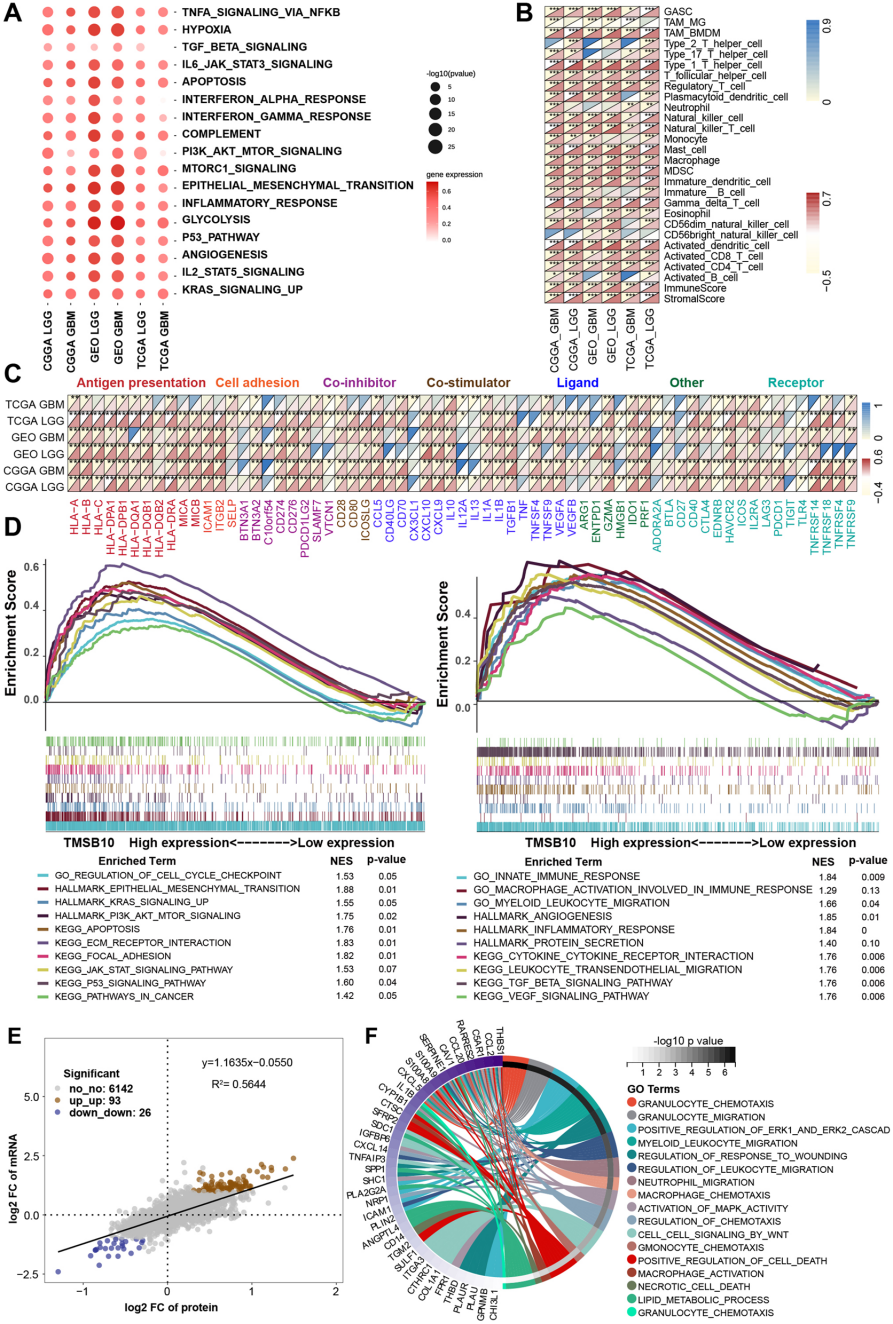
Biological pathways and immunological characteristics of TMSB10 in glioma. **A** Spearman correlation analysis of TMSB10 and classical signaling pathways in three glioma cohorts. Red indicates positive correlations, and the darkness of color is proportional to the correlation coefficient. The size of the circle represents the statistical P value, with larger circles representing greater statistical significance. Correlation between TMSB10 expression and **B** the infiltration of 28 tumor-associated TME cells calculated with the ssGSEA algorithm and **C** immunomodulators. The color indicates the correlation coefficient. The asterisks indicate a statistically significant p value calculated using Spearman correlation analysis. **D** GSEA showing the (left) classical cancer-promoting pathways and (right) immune-related pathways in the high TMSB10 expression group. **E** Dot plot of the log2FC (mRNA expression) versus the log2FC (protein expression), showing a positive correlation between the overall mRNA and protein expression level (Pearson’s r = 0.6527) and the distribution of genes with significant changes in both the mRNA expression (|FC| > 2, P < 0.05) and corresponding protein expression (|FC| > 1.2, P < 0.05) in the high TMSB10 expression group compared with the low TMSB10 expression group. **F** GO BP enrichment analysis of 93 genes that were significantly upregulated at both the mRNA and protein levels. The statistical significance is shown as P < 0.05; **P < 0.01; ***P < 0.001

To further investigate the biological function of TMSB10 in glioma, we performed gene set variation analysis (GSVA) to identify cancer hallmarks that are closely associated with TMSB10 expression. The results showed that carcinogenic processes such as inflammatory responses, stromal activation, cell proliferation and invasion-related pathways were positively correlated with TMSB10 expression in both LGG and GBM in these three glioma datasets (Fig. 4A). Subsequent analysis of TME cell infiltration, calculated by the single-sample gene set enrichment analysis (ssGSEA) algorithm, showed that TMSB10 was positively correlated with the abundance of most immune cells (Fig. 4B). In addition, TMSB10 was also positively correlated with the expression of most immunomodulatory molecules in these three glioma datasets (Fig. 4C). We also showed similar results in the other three GBM datasets (Additional file 1: Fig. S4B, C). Gene set enrichment analysis (GSEA) showed that pathways involved in tumor pathogenesis, including cell proliferation regulation pathways (such as cell cycle checkpoint and apoptosis), pathways associated with tumor invasion and migration (such as focal adhesion and ECM-receptor interaction), and classical signaling pathways, including p53 signaling and PI3K AKT mTOR signaling pathways and immune-related signatures, were significantly enriched in the high TMSB10 expression group (Fig. 4D). In addition, Metascape database [19] analysis revealed that genes with a significant positive association with TMSB10 (Additional file 2: Table S1, Pearson r > 0.3, P < 0.05) were significantly enriched in pathways related to the regulation of cell biological functions, stromal activation and immunity (Additional file 1: Fig. S4D). Further PaGenBase [20] enrichment analysis showed that these genes were mainly specifically expressed in peripheral immune organs such as the spleen, blood and bone marrow (Additional file 1: Fig. S4E).

Further joint analysis of the protein and RNA-seq data revealed a positive correlation between the differentially expressed mRNAs and proteins; 93 mRNA/protein were simultaneously upregulated, and 26 mRNA/protein were simultaneously downregulated (Fig. 4E, Additional file 2: Table S2). Kyoto Encyclopedia of Genes and Genomes (KEGG) functional enrichment analysis showed that the genes upregulated at both levels were mainly enriched in some classical oncogenic pathways, metabolic pathways and immune response-related pathways (Additional file 1: Fig. S5A). Further enrichment analysis by gene ontology (GO) database showed that these genes were significantly enriched in the following terms: immune cell migration, angiogenesis, stromal remodeling, macrophage activation and ERK signaling pathways (Fig. 4F). Overall, these results revealed that TMSB10 could be a potential biomarker for alterations in TME cell infiltration and carcinogenic pathways in glioma.

### Multiomics regulatory profile of TMSB10 in glioma

**Fig. 5:**
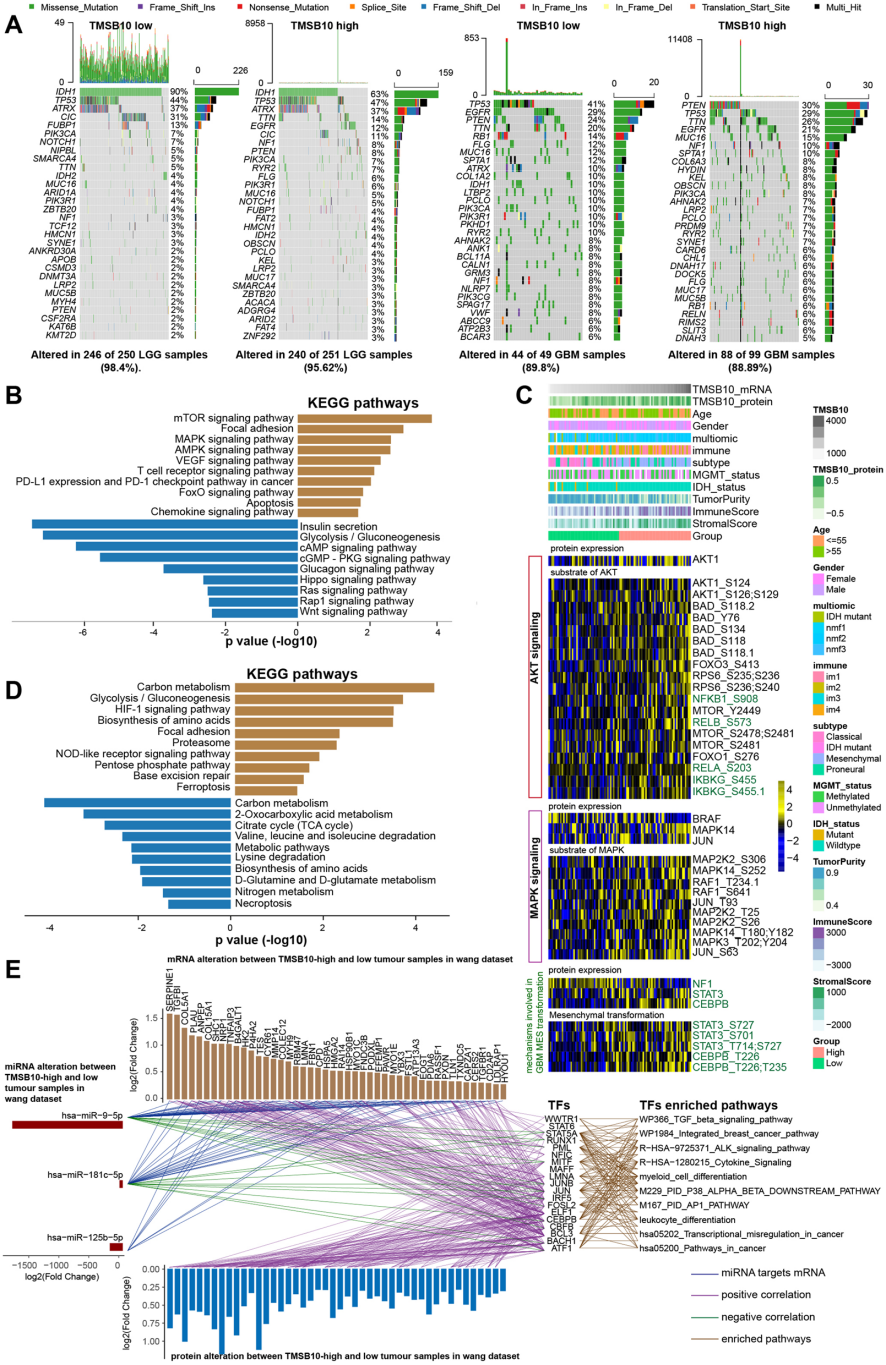
Multiomics regulatory profile of TMSB10 in glioma. **A** waterfall plot of the tumor somatic mutation landscape in the low-TMSB10 and high-TMSB10 samples in the TCGA-LGG (left) and GBM (right) datasets. Each bar represents the mutation information for an individual patient. The top bar plot shows TMB, and the numbers on the right indicate the mutation frequency of each gene. The bar plot on the right shows the proportion of each mutation type. **B** KEGG enrichment analysis of proteins with significantly different phosphorylation levels in GBM samples with high TMSB10 expression. The right represents the upregulated pathway, and the left represents the downregulated pathway. **C** Heatmap showing somatic mutation-based alterations in specific proteins and their downstream protein phosphorylation sites. Yellow represents a high level, and blue represents a low level. **D** KEGG enrichment analysis of proteins with significantly different acetylation levels in GBM samples with high TMSB10 expression. The right represents the upregulated pathway, and the left represents the downregulated pathway. **E** Interaction of TMSB10-associated miRNAs, mRNA, protein and transcription factor (TF). Upregulated mRNAs (claybank bars in the upper panel) and proteins (blue bars in the lower panel) in TMSB10-high GBM were positively correlated (purple lines) with the expression of 19 TFs (Pearson R > 0.5, p < 0.05). Downregulated miRNAs (red bars, left panel) negatively correlated (green lines) with TF expression (Pearson R< − 0.3, p < 0.05). The targeted pathways of TFs are listed in the panel to the right. The blue line indicates miRNA-targeted mRNAs

To better understand the determinants of glioma evolution and treatment resistance, we then analyzed the differences in somatic mutation distribution between the high and low TMSB10 expression groups in the TCGA-LGG and GBM cohorts and found that the rate of IDH mutation (low: 90%, high: 63%), a factor associated with a better prognosis, was obviously decreased in the high-TMSB10 group compared to the low-TMSB10 group in the TCGA-LGG cohort. For GBM samples, the mutation rates of PTEN (low: 24%, high: 30%) and NF1 (low: 8%, high: 10%) were notably higher in the high expression group than in the TMSB10 low expression group (Fig. 5A). Similar results were also shown in another GBM dataset. Additionally, BRAF1 mutation, which causes activation of the downstream MAPK signaling pathway, also occurred at a higher rate in the high expression group (Additional file 1: Fig. S5B).

Exploring the panoramic map of phosphorylomic and acetylationomic differences between the high and low TMSB10 expression groups may provide more valuable clues to explore the mechanism of malignant progression of glioma. The analysis of differentially phosphorylated proteins showed that phosphorylation was markedly upregulated at 1146 phosphorylation sites of 590 proteins and markedly downregulated at 498 phosphorylation sites of 265 proteins in the high-TMSB10 group (Additional file 2:  Table S3, |FC| > 1.2, adj.P.Val < 0.05). KEGG enrichment analysis showed that proteins with upregulated phosphorylation were significantly enriched in oncogenic signaling pathways, stromal activation pathways and immune-related signaling pathways, while downregulated proteins were mainly involved in metabolism-related pathways, the Rap1 signaling pathway, and so on (Fig. 5B). Further analysis of the combined protein quantification data showed a significant positive correlation between phosphorylation levels and protein expression levels (Additional file 1: Fig. S5C, Additional file 2: Table S4). Analysis of the mutation distribution in the TCGA and Wang datasets showed that the PTEN (which inhibited AKT signaling activation) and NF1 (a mesenchymal (MES) subtype GBM marker) mutation rates were increased in both datasets, and BRAF (which could cause MAPK downstream pathway activation) mutation rates were obviously increased in the Wang dataset (Fig. 5A, Additional file 1: Fig. S5 A). We next explored the specific signaling pathways induced by these three mutations and the altered phosphorylation of their downstream proteins. As shown in the heatmap, the expression levels of the tumor suppressor proteins NF1 and BRAF tended to decrease with increasing TMSB10 mRNA expression, and we also observed a gradual increase in the phosphorylation levels of downstream signaling pathway proteins (Fig. 5C). These results support that TMSB10 contributes to the development of GBM through the dysregulation of protein expression and protein phosphorylation.

The proteome-level differential acetylation analysis showed that compared to the low-TMSB10 group, the high group showed significantly upregulated acetylation at 139 acetylation sites in 93 proteins and significantly downregulated acetylation at 38 acetylation sites in 28 proteins (Additional file 2: Table S5, FC > 1.2, adj.P.Val < 0.05). KEGG functional annotation analysis showed that proteins with different acetylation levels were mainly enriched in metabolic-related signaling pathways, HIF-1 signaling pathways and immune-related signaling pathways (Fig. 5D). Further correlation analysis with proteomics data also showed that the acetylation levels were positively correlated with the protein expression levels (Additional file 1: Fig. S5D, Additional file 2: Table S6).

To further investigate the effect of TMSB10 on miRNA expression, we identified 3 differentially downregulated miRNAs (miR-9-5p, miR-181c-5p and miR-125b-5p) in high TMSB10 and low-TMSB10 GBM samples in the Wang dataset. The mRNA and protein expression levels of multiple target genes of these three miRNAs were significantly upregulated in the TMSB high group. Further correlation and functional enrichment analysis revealed multiple transcription factors (TFs) that were negatively correlated with the expression of these three miRNAs, positively correlated with the expression of their target genes, and significantly enriched in immune regulation, stromal activation and MAPK signaling pathways (Fig. 5E). Taken together, our findings suggest that TMSB10 affects the regulatory network of glioma at multiple levels, providing a comprehensive overview of how TMSB10 affects glioma pathogenesis.

### **TMSB10 expression is associated with glioma pathological malignant progression and immune phenotype in the Qilu dataset**

**Fig. 6:**
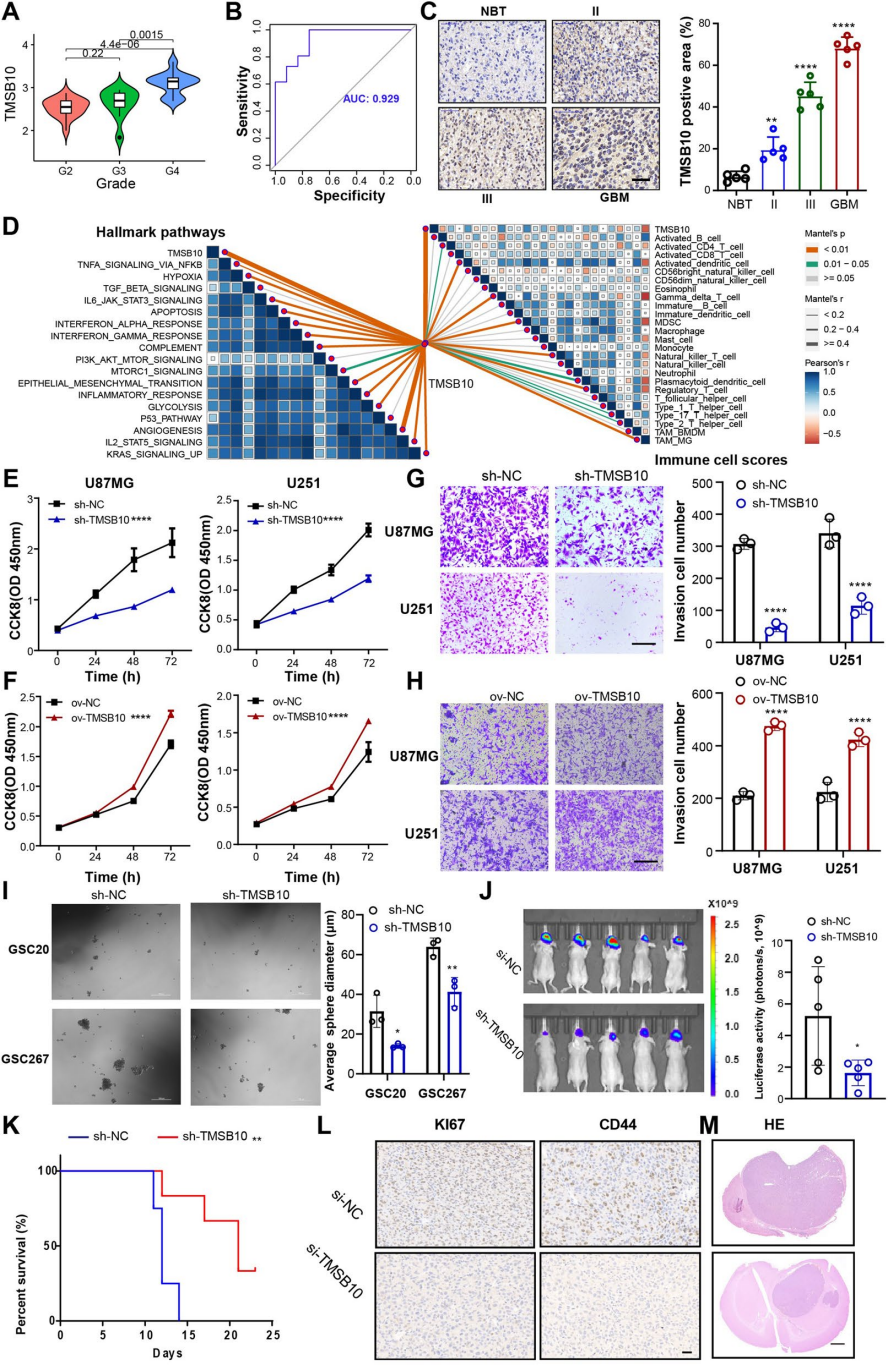
TMSB10 promotes the proliferation, migration and invasion of glioma cells in vitro and in vivo. **A** The mRNA expression level of TMSB10 increased with tumor grade in the Qilu dataset. **B** ROC curve showing that the expression level of TMSB10 had a high AUC in predicting the grade of glioma in the Qilu dataset. **C** IHC analysis showing TMSB10 protein expression in normal brain tissues (NBTs) and glioma tissues with different WHO grades. Histogram representing statistical data of IHC. The FTO-positive ratio was defined as the ratio of the FTO-positive area to the total area; n = 3. **D** Correlations between TMSB10 and the enrichment scores of (left) cancer hallmark pathways and (right) immune cells in the Qilu cohort. CCK-8 assays showing the proliferation ability of GBM cells transfected with (**E**) sh-NC or sh-TMSB10 and (**F**) ov-NC or ov-TMSB10; n = 3. G Representative Transwell migration and invasion assays showing the migration and invasion ability of GBM cells transfected with (**G**) sh-NC or sh-TMSB10 and (**H**) ov-NC or ov-TMSB10; scale bar, 200 μm. The quantification histogram represents the relative cell numbers. Data represent the mean ± SD from at least three independent experiments. **I** Representative tumor sphere formation images of GSCs transfected with sh-NC or sh-TMSB10; scale bar, 100 μm. The quantification histogram represents the average sphere diameter. Data represent the mean ± SD from at least three independent experiments. **J** Bioluminescent image showing the tumor size of mice implanted with luciferase-labeled U87MG cells expressing sh-TMSB10 or sh-NC at the indicated times. The quantification histogram represents the bioluminescent flux. Data represent the mean ± SD; n = 5 for each group. **K** Kaplan–Meier survival curves for mice implanted with luciferase-labeled U87MG cells expressing sh-TMSB10 or sh-NC. Log-rank analysis was used; n = 5 for each group. **L** Representative CD44 and KI67 immunohistochemistry images for a subgroup of animals sacrificed simultaneously in each group; n = 5 for each group, scale bar, 20 μm. **M** Representative H&E staining images for a subgroup of animals sacrificed simultaneously in each group; n = 5 for each group, scale bar, 10 μm. All data are presented as the mean ± SD. The statistical significance is shown as *P < 0.05; **P < 0.01; ****P < 0.0001

In our cohort, TMSB10 positively correlated with glioma grade, and the results showed that the GBM samples had the highest TMSB10 expression (Fig. 6A). ROC analysis for TMSB10 expression and glioma grade showed that the AUC was 0.929 (Fig. 6B), suggesting that TMSB10 has the potential to serve as a biomarker for predicting glioma grade. Furthermore, IHC analysis of our local glioma clinical samples validated that TMSB10 expression was highest in GBM samples (Fig. 6C), consistent with the results of the database analyses. To explore the relationship between biological functions and TMSB10, GSVA analysis was conducted using the glioma samples in our cohort. The results showed that the TMSB10-high group demonstrated a marked enrichment of immune-related pathways, stromal activation pathways, and pathways in cancer (Additional file 1: Fig. S6A). GSEA also showed that these functional gene set signatures were enriched in TMSB10-high glioma samples (Additional file 1: Fig. S6B). For TME immune regulation in our cohort, we found that TMSB10-high group samples have higher immune/stromal score, lower tumor purity, and remarkably higher innate immune cell scores (Additional file 1: Fig. S6C). Further Pearson correlation analysis also showed that TMSB10 was positively correlated with these cancer hallmarks and related immune cell scores (Fig. 6D), paralleling the above results. We then performed Pearson correlation analysis to identify the genes that tightly correlated with TMSB10 expression (Pearson |R| > 0.3, P < 0.05 Additional file 2: Table S7). Further gene enrichment analysis using Cytoscape dayabase revealed that genes that were significantly positively associated with TMSB10 were significantly enriched in immune-related, stromal activation-related and cancer malignant progression-related signaling pathways (Additional file 1: Fig. 6D). Further PaGenBase database analysis also showed that these genes were mainly specifically expressed in peripheral immune organs such as the spleen, blood and bone marrow (Additional file 1: Fig. S6E), similar to the above results.

### TMSB10 promotes the proliferation, migration and invasion of glioma cells in vitro and in vivo

To validate the function of TMSB10 in glioma proliferation, metastasis and invasion, we performed several related experiments in vitro and in vivo. We assessed the TMSB10 expression level of the two primary cell lines and other cell lines and found there was no remarkable difference among these cell lines (Additional file 1: Fig. S8D). The results showed that knockdown of TMSB10 significantly inhibited the proliferation of U87MG and U251 GBM cells (Fig. 6E, Additional file 1: Fig. S7A–C), as detected by Cell Counting Kit-8 (CCK-8), colony formation and EdU assays. Overexpression of TMSB10 remarkably promoted these cellular behaviors in GBM cells (Fig. 6F, Additional file 1: Fig. S7D). Moreover, Transwell and wound healing assays showed that knockdown of TMSB10 impaired the migration and invasion abilities of U87MG and U251 GBM cells (Fig. 6G, Additional file 1: Fig. S7E), while overexpression of TMSB10 notably promoted these cellular behaviors (Fig. 6H). Western blot results also showed that knockdown of TMSB10 increased the expression of P21 (a tumor suppressor protein) but decreased the expression of carcinogenic proteins involved in cell proliferation and invasion and the result was consistent in mice tumor samples (Additional file 1: Fig. S7F). Flow cytometric analysis showed that TMSB10 knockdown could induce apoptosis.and G1/S arrest in glioma cells (Additional file 1: Fig. S8A–C). Glioma stem cells (GSCs) are considered to be the drivers of GBM growth and progression [21]. We then performed neurosphere formation assays of GSC20 and GSC267 GSCs, which showed that knockdown of TMSB10 significantly inhibited the tumorsphere expansion ability (Fig. 6I).

Additionally, in vivo experiments also showed that the downregulation of TMSB10 significantly reduced tumor size and prolonged the survival time of tumor-bearing mice (Fig. 6J, K). Subsequently, immunohistochemical (IHC) results showed that compared with the NC group, the expression of Ki67 (proliferation marker) and CD44 (invasion marker) in tumor tissue sections was lower in the TMSB10 knockdown group (Fig. 6L). H&E staining also showed that the invasive ability of tumors following TMSB10 knockdown was weaker than that of the NC group (Fig. 6M). In conclusion, these results suggested that TMSB10 promotes cell proliferation, migration and invasion, playing oncogenic roles in glioma.

### TMSB10 promotes GBM MES transformation and facilitates macrophage infiltration

**Fig. 7:**
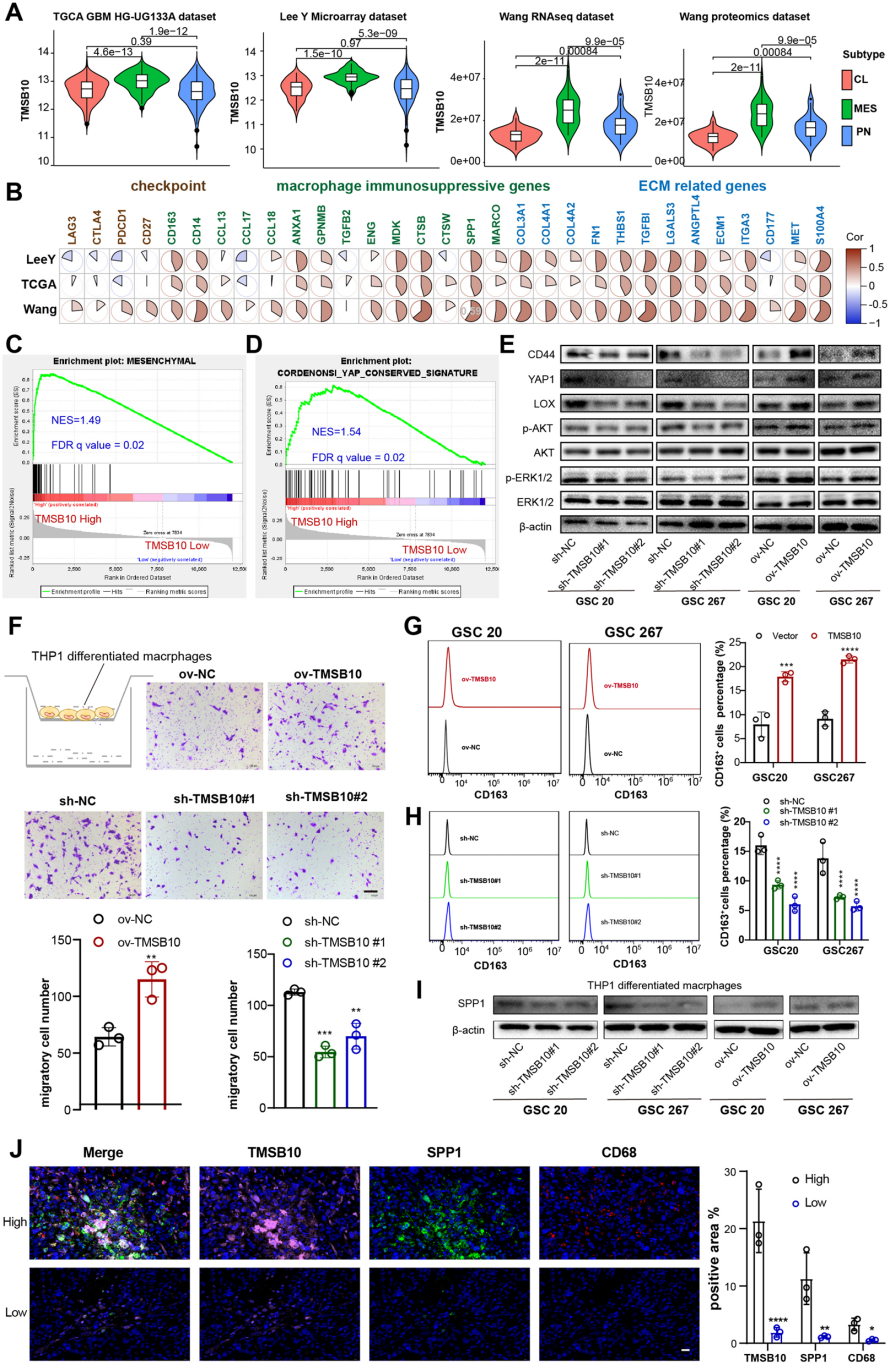
TMSB10 promotes GBM MES transformation and facilitates macrophage infiltration. **A** The mRNA or protein expression level of TMSB10 in different subtypes of GBM. **B** Correlation between TMSB10 and suppressive immunomodulators. The color indicates the correlation coefficient. GSEA showing the **C** MES signature and **D** CORDENONSI_YAP_CONSERVED_SIGNATURE in the high TMSB10 expression group in the TCGA GBM cohort. **E** Western blot assays showing the protein expression of CD44, YAP1, LOX, as well as phosphorylation levels of AKT and ERK1/2 in GSCs transfected with sh-NC or sh-TMSB10 and ov-NC or ov-TMSB10 as indicated. **F** (Top) Representative Transwell migration assays showing the chemotaxis capacity of human THP-1-differentiated macrophages by exposing them to CM from GSCs transfected with sh-NC or sh-TMSB10 and ov-NC or ov-TMSB10 as indicated. (Bottom) Quantification histogram representing relative cell numbers; n = 3, scale bar, 100 μm. **G** Representative flow cytometry histogram showing the proportion of CD163 + in THP-1 differentiated macrophages treated with CM from GSCs transfected with **G** sh-NC or sh-TMSB10 and **H** ov-NC or ov-TMSB10 as indicated. The quantification histogram represents the proportion of CD163 + differentiated THP-1 macrophages; n = 3. **I** Western blot assays showing the protein expression of SPP1 in THP-1 differentiated macrophages treated with CM from GSCs transfected with sh-NC or sh-TMSB10 and ov-NC or ov-TMSB10 as indicated. **J** Representative IF staining in a human glioma tissue microarray showed that the expression of SPP1 and CD68 was higher in the TMSB10-high group than in the TMSB10-low group. Histogram representing statistical proportion data of positive area. All data are presented as the mean ± SD. The statistical significance is shown as *P < 0.05; **P < 0.01; ***P < 0.001; ****P < 0.0001

The TCGA program uses bulk RNA sequencing to identify GBM into three subtypes: proneural (PN), classical (CL) subtypes and MES subtypes. The MES subtype, accompanied by NF1 mutations, which are more common among TMSB10-high glioma samples (Fig. 5A, Additional file 1: Fig. S5A), was correlated with an abundance of macrophages, stromal activation and the worst prognosis [22, 23]. Our data showed that both the mRNA and protein expression of TMSB10 was highest in the MES GBM subtype in all three GBM datasets (Fig. 7A). Pearson correlation analysis also showed that TMSB10 was positively correlated with immune checkpoints, macrophage-related immunosuppressive molecules and stromal-related genes in six public glioma datasets and the Qilu dataset (Fig. 7B, Additional file 1: Fig. S8A, B). Further correlation analysis also showed that TMSB10 was significantly positively correlated with CD44 expression, a MES marker gene (Additional file 1: Fig. S8B). Additionally, GSEA showed that the MES gene signature was significantly enriched in TMSB10-high GBM samples (Fig. 7C, Additional file 1: Fig. S8A), implying that TMSB10 may promote the MES transformation of GBM. Chen et al. [24] found that PTEN deficiency, which was also upregulated in TMSB10-high GBM samples, could increase macrophage infiltration via the YAP1-LOX-β1 integrin-PYK2 axis, and the infiltrated macrophages in turn secreted SPP1 to support GBM survival. Our GSEA also showed that YAP1 signaling (CORDENONSI_YAP_CONSERVED_SIGNATURE) was significantly enriched in the TMSB10-high samples (Fig. 7B, Additional file 1: Fig. S8C). Furthermore, our Western blot assays verified that TMSB10 promoted the protein expression of MES-related genes and YAP1 and activated AKT and MAPK signaling (Fig. 7C), consistent with the above results, prompting us to conclude that TMSB10 may be associated with the infiltration and immunosuppressive transformation of macrophages. TAMs, including tumor-associated bone marrow-derived macrophages (BMDMs) and brain-resident microglial cells (MGs), are the most abundant cell population in the glioma TME, [25, 26]. We found that TMSB10 was positively correlated with the enrichment of tumor-associated BMDMs (hereinafter referred to as macrophages) but negatively correlated with the enrichment of tumor-associated MGs (Figs. 5B and 6D), suggesting that TMSB10 is involved in the recruitment of macrophages. To further confirm the role of TMSB10 in promoting macrophage migration and immunosuppressive transformation, we found that conditioned medium (CM) from TMSB10-overexpressing GBM cells significantly promoted THP-1-differentiated macrophage migration and significantly upregulated the protein expression of CD163 and SPP1, two well-known macrophage activation markers [24, 26–28], compared to that in the NC group, while knockdown of TMSB10 significantly inhibited these effects (Fig. 7D–G). Moreover, we then performed an immunofluorescence staining assay with our local clinical glioma patient tissues, and the results showed that the expression of CD68 (a human macrophage marker) and SPP1 (a key inflammatory protein produced by TAMs that express the pattern recognition receptor MARCO and part of the largest group of proteins with cell type-specific expression) [29], was enhanced in TMSB10-high glioma tissues compared to TMSB10-low tissues (Fig. 7H). Overall, these results suggested that TMSB10 promotes macrophage infiltration, thereby enabling macrophages to acquire angiogenic and immunosuppressive properties, highlighting its potential as a therapeutic target.

### TMSB10 could be a potential immunotherapy target for glioma patients, and knockdown of TMSB10 improves the efficacy of selumetinib and anti-PD1 treatment in glioma

**Fig. 8:**
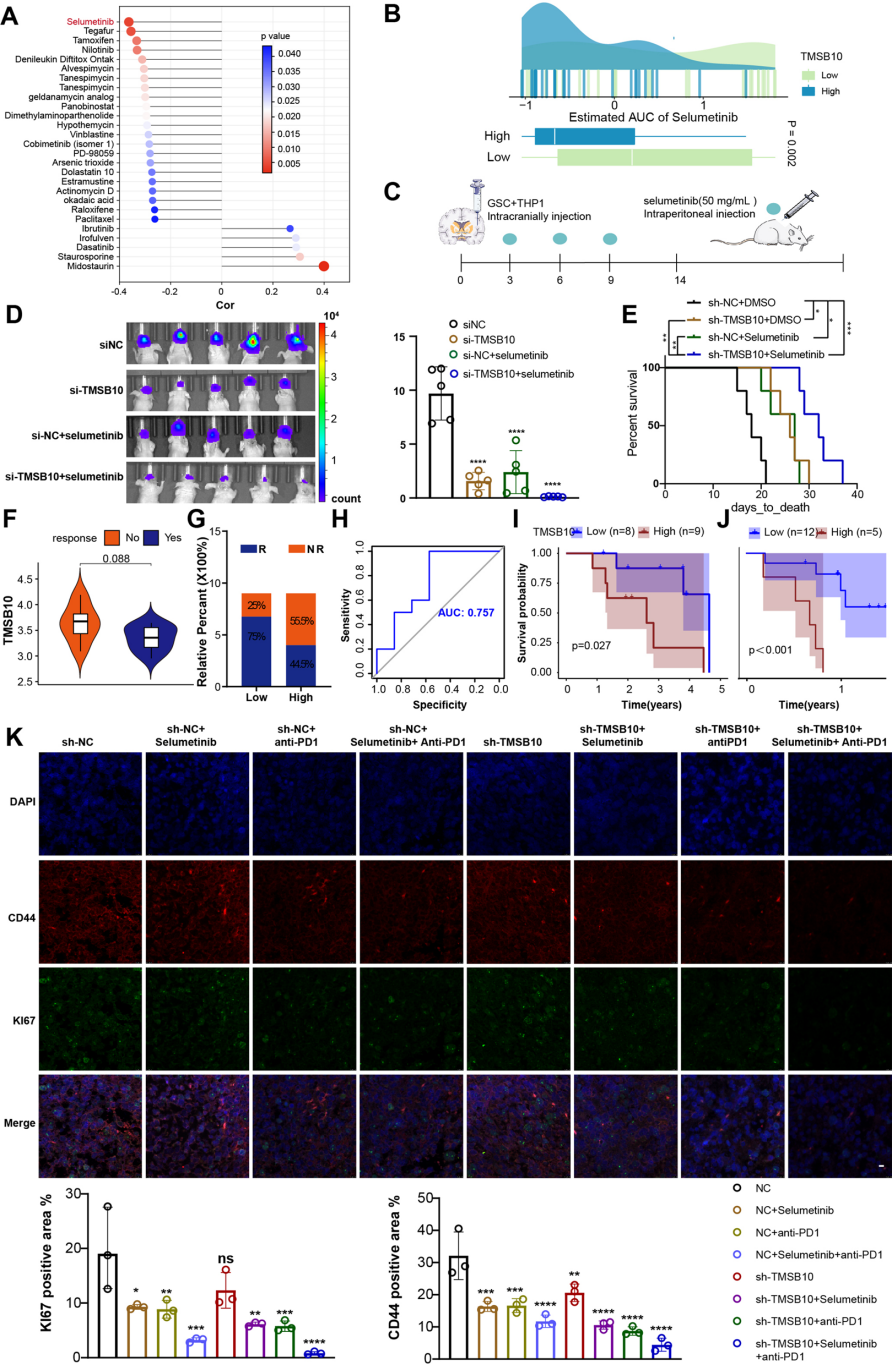
TMSB10 could be a potential immunotherapy target for glioma patients, and knockdown of TMSB10 improves the efficacy of selumetinib and anti-PD1 in glioma. **A** Spearman’s correlation analysis between TMSB10 and the bioavailability (AUC) of the drugs that have undergone clinical trials or received FDA approval was collected from the CellMiner database (https://discover.nci.nih.gov/cellminer/). **B** Comparison of the estimated selumetinib AUC between the TMSB10-high group and the TMSB10 low group. **C** Animal experiment protocol design process. **D** Bioluminescent image showing the tumor size of mice coimplanted with THP-1 cells and luciferase-labeled GSC267 expressing sh-TMSB10 or sh-NC and treated with selumetinib (50 mg/ml) for the indicated times. The quantification histogram represents the bioluminescent flux. Data represent the mean ± SD; n = 5 for each group. **E** Kaplan–Meier survival curves showing the mice coimplanted with THP-1 cells and luciferase-labeled GSCs expressing sh-TMSB10 or sh-NC and treated with selumetinib (50 mg/ml). Log-rank analysis was used; n = 5 for each group. **F** Expression of TMSB10 in distinct anti-PD1 clinical response groups. **G** The proportion of patients who responded to anti-PD1 immunotherapy in the low or high TMSB10 expression groups. R, response; NR, no response. H ROC curve quantifying the predictive value of TMSB10 in GBM patients treated with anti-PD1 therapy (AUC, 0.757). Kaplan–Meier curves for (**I**) the OS of GBM patients (log-rank test P = 0.027) and **J** survival duration after anti-PD1 treatment (log-rank test P < 0.001 in the PD1 dataset. **K** GBOs at 1 week were cocultured with CM from GSC 267 cells, transfected with sh-TMSB10 or sh-NC, and treated with PD1 antibody (5 µM) and/or selumetinib (7.5 µM) for 5 days as indicated. IF staining for KI67 and CD44 in GBO sections showed that knockdown of TMSB10 enhanced the effect of anti-PD1 and selumetinib therapy; scale bars: 10 μm. Histogram representing statistical data for proportion of positive area; n = 3 for each group. All data are presented as the mean ± SD. The statistical significance is shown as follows: ns > 0.05; *P < 0.05; **P < 0.01; ***P < 0.001; ****P < 0.0001

To explore potential drugs associated with TMSB10, we obtained data regarding the bioavailability (AUC) of drugs that have undergone clinical trials or received FDA approval from the CellMiner database (https://discover.nci.nih.gov/cellminer/) and assessed the correlation of bioavailability with TMSB10 expression. Using Spearman’s correlation analysis, we identified 27 drugs significantly associated with TMSB10 (Fig. 8A, Additional file 2: Table S8). The AUC of selumetinib, a mitogen-activated protein kinase 1 and 2 (MEK1/2) inhibitor approved by the FDA for the treatment of tumors associated with neurofibromatosis [30], had the highest negative correlation with the expression of TMSB10 (Fig. 8A). We found that compared to that in the low expression group, the AUC of selumetinib was significantly lower in the TMSB10 high expression group (Fig. 8B), suggesting that TMSB10 may decrease the sensitivity of tumor cells to this drug. To further verify the effect of TMSB10 knockdown on the immunosuppressive polarization of macrophages in vivo and to determine whether the combination of a TMSB10-targeted approach with selumetinib can improve the therapeutic effect on GBM, GSCs with TMSB10 knockdown or NC vectors were coimplanted with THP-1 cells in situ in the brains of nude mice, followed by treatment with or without selumetinib. We found that compared to the NC group, the TMSB10 knockdown group displayed decreased tumor growth and prolonged survival of tumor-bearing mice. Downregulation of TMSB10 in combination with selumetinib presented the best therapeutic results (Fig. 8C–E).

Zhao J and his colleagues longitudinally profiled 66 patients, including 17 long-term responders, during standard therapy and after treatment with PD-1 inhibitors (nivolumab or pembrolizumab) and revealed significant enrichment of PTEN mutations associated with immunosuppressive expression signatures and significantly higher PI3K-AKT pathway activity among PTEN-mutant nonresponsive tumors [31]. Our results also showed that the PTEN mutation rate and PI3K-AKT pathway activity were upregulated in TMSB10-high glioma samples (Fig. 4A, D, and Fig. 5A, C). In addition, TMSB10 expression showed a significant positive correlation with immune checkpoint expression in glioma patients (Fig. 4C, Additional file 1: Fig. S8E, F). Intriguingly, antibody-mediated MARCO targeting promotes the M1-like polarization of TAMs and enhances immune checkpoint inhibitor (ICI) efficacy [32], indicating that knockdown of TMSB10 may enhance the efficacy of ICIs in GBM. We then evaluated the ability of TMSB10 expression level to predict patient response to ICI therapy in a GBM dataset of patients who received PD1 inhibitor therapy [31]. We found that the low-TMSB10 group exhibited better therapeutic benefit and a better clinical response to PD1 inhibitor immunotherapy than the high-TMSB10 group (Fig. 8F, G). ROC curve analysis showed that TMSB10 had good predictive power in terms of the effectiveness of immunotherapy in GBM patients (Fig. 8H, AUC = 0.757). Further survival analysis revealed that patients with low TMSB10 expression showed a significant clinical advantage and significantly prolonged survival, as measured from the start of diagnosis or treatment with anti-PD1 therapy (Fig. 8I, J).

To further explore the therapeutic efficacy of knocking down TMSB10 in combination with selumetinib and PD1 inhibitor treatment for GBM, we generated a patient-derived glioblastoma organoid (GBO) that maintained the heterogeneity of cell types (including macrophages, T cells, and vascular cells) and the molecular characteristics of their respective parental tumors [33] as a model for holistic study of the TME of tumors and evaluating the efficacy of PD1 inhibitors [34, 35]. We exposed GBO samples from 1 patient, whose GBM tissue expressing high TMSB10, CD68 and SPP1, to CM from TMSB10 knockdown GSCs at the indicated time, and then treated the GSCs in combination with PD1 inhibitor (5 µM) and/or selumetinib (50 mg/ml) for 5 days. We evaluated the therapeutic response by quantifying the percentage of cells expressing Ki67 (a proliferation marker) and CD44 (an invasion marker), showing that the downregulation of TMSB10 significantly improved the efficacy of PD1 inhibitors as well as selumetinib in GBM, and the combination of the three treatment strategies was the most effective (Fig. 8K). Collectively, these results highlighted that TMSB10 may serve as a potential prognostic biomarker and immunotherapy target in glioma, as its knockdown significantly improved the efficacy of selumetinib and anti-PD1 treatment, providing a promising strategy for improving the response to targeted combination therapy for glioma patients.

## Discussion

Mounting research suggests that TMSs may play an important role in the malignant progression and clinical diagnosis of cancer [36–39]. In this study, we comprehensively investigated the basal expression levels of TMSs in human normal tissues, as well as their genomic mutation, CNA, expression dysregulation, prognosis and immunotherapeutic response implications across 33 human cancer samples. Our results showed that TMSs were differentially expressed in numerous cancer types. In the genomic landscape, we found that the overall DNA alteration frequency of TMSs was less than 2%, which is relatively low (Fig. 1B, C). We found that TMSs were significantly associated with patient survival in various cancers and that TMSB10 could act as a prognostic risk factor for nine tumors (Fig. 1D). Further analysis of the expression patterns of TMSs in normal and tumor tissues revealed that TMSB4Y, TMSB15A and TMSB15B were expressed at low levels, while other genes were expressed at relatively high levels; TMSB10 was expressed at the highest level and was also specifically overexpressed in almost all types of tumor tissues verus normal tissues (Fig. 1H, I), suggesting that TMSB10 might play a pivotal role in cancer development.

We further comprehensively evaluated the expression pattern, biological function and immunological role of TMSB10 across 33 cancers. Strikingly, we found that TMSB10 was specifically overexpressed in glioma tissues and was closely associated with glioma prognosis, biological function, and immune regulation (Fig. 2). We obtained similar results in multiple other public glioma datasets and our Qilu local dataset, validating that TMSB10 could distinguish glioma and normal brain tissues (Figs. 3 and 4). Additionally, the immunohistochemical results for our local glioma tissues further validated that TMSB10 expression levels were higher in glioma tissues than in normal tissues and were upregulated with increasing grade. Our study further provided a comprehensive multiomics view of TMSB10 in glioma, including changes in mRNA, miRNA, protein expression, phosphorylation and acetylation, as alterations at these levels might affect a wide range of biological processes, including inflammatory responses, angiogenesis, apoptosis, and other pro-oncogenic signaling pathways (Fig. 5). These results suggest that TMSB10 leads to molecular changes at multiple levels, thereby playing a key role in cancer development.

To further demonstrate the pro-carcinogenic function of TMSB10 in glioma, we performed several related experiments in vitro and in vivo. Our results suggested that TMSB10 promotes cell proliferation, migration and invasion, thereby playing oncogenic roles in glioma (Fig. 5). Our data also showed that TMSB10 might be involved in immune regulation. The imbalance of the TME may also partly contribute to the poor prognosis associated with TMSB10 in glioma. Macrophages are the main nontumor cells that infiltrate the glioma microenvironment. Studies have shown that macrophages can alter their expression profiles based on the signals they receive from tumor cells; thus, GBM heterogeneity leads to macrophage heterogeneity. Similarly, macrophages can also shape the expression profile of tumor cells, which in turn drives the MES transformation of glioma. Further integration with other biological experiments also revealed the key roles of TMSB10 in the MES transformation of glioma, the promotion of macrophage infiltration and immunosuppressive polarization (Fig. 7).

We also confirmed the good performance of TMSB10 in the prediction of the anti-PD1 immunotherapy response. We also identified multiple drugs targeting high TMSB10 expression and validated that knockdown of TMSB10 improved the efficacy of selumetinib (a MEK1/2 inhibitor approved by the FDA for the treatment of neurofibromatosis-associated tumors) and anti-PD1 in glioma (Fig. 8), providing a promising strategy for improving targeted combination therapy for glioma patients.

## Conclusion

In conclusion, our data provide a landscape of TMSs in cancer. We highlighted that TMSB10 may serve as a potential prognostic biomarker and immunotherapy target in glioma and that the knockdown of TMSB10 significantly improves the efficacy of selumetinib and anti-PD1 treatment, providing a theoretical basis for the development of more effective and targeted clinical treatment strategies for glioma patients. The proportions of CAFs were estimated by applying the microenvironment cell population counter (MCP-Counter) method.

## Supplementary Information

**Additional file 1: Figure S1.** The expression of TMSs in cancer cell lines in CCLE dataset. The expression of **(A)** TMSB10, **(B)**TMSB4X, **(C)** TMSB4Y, **(D)** TMSB15A, **(E)** TMSB15B, **(F)** PTMA and **(G)** PTMS in cancer cell lines in CCLE dataset. **Figure S2.** The expression of TMSs in normal tissues in GETx dataset. The expression of **(A)** TMSB10, **(B)**TMSB4X, **(C)** TMSB4Y, **(D)** TMSB15A, **(E)** TMSB15B, **(F)** PTMA and **(G)** PTMS in normal tissues in GETx dataset. **Figure S3**. The expression of TMSs between GTEx normal tissues and paired tumor tissues. The expression of **(A)**TMSB4X, **(B)** TMSB4Y, **(C)** TMSB15A, **(D)** TMSB15B, **(E)** PTMA and **(F)** PTMS between GTEx normal tissues and tumor tissues. The asterisks indicated a statistically significant p-value calculated using Mann-Whitney U test. The statistical significance is indicated as follows: ns>0.05; *P < 0.05; **P < 0.01; ***P < 0.001. **Figure S4.** Biological pathways and immunological characteristics of TMSB10 in glioma. **A **Kaplan–Meier curves for the OS of GBM patients with high TMSB10 expression and low TMSB10 expression in three GBM datasets; the log-rank test was used to calculate the p value. **B** Correlation between TMSB10 and the infiltration of 25 tumor-associated TME cells calculated with the ssGSEA algorithm. The color indicates the correlation coefficient. **C** Spearman correlation analysis of TMSB10 and classical signaling pathways in three glioma cohorts. Red indicates positive correlations, and the darkness of color is proportional to the correlation coefficient. The size of the circle represents the statistical P value, with larger circles representing greater statistical significance. Bar graph of (**D**) enriched terms, colored by p-values, and (**E**) summary of enrichmentanalysis in TRRUST across genes positively correlated with TMSB10 in CGGA glioma dataset. **Figure S5.** Multiomics regulatory profile of TMSB10 in glioma. **A **KEGG enrichment analysis of genes with significantly upregulated at both mRNA and protein levels in GBM samples with high TMSB10 expression. **B** Waterfall plot of the tumor somatic mutation landscape in the (upper) low-TMSB10 and (lower) high-TMSB10 samples in the wang GBM dataset. Dot plot of the log2FC (protein expression) versus the (**C**) log2FC (protein phosphorylation expression), and (**D**) log2FC (protein acylation expression), showing a positive correlation between the overall protein phosphorylation/acylation level and protein expression and the distribution of genes with significant changes in both the protein expression (|FC| > 1.2, P < 0.05) and corresponding protein phosphorylation/acylation expression (|FC| > 1.2, P < 0.05) in the high TMSB10 expression group compared with the low TMSB10 expression group. **Figure S6.** TMSB10 expression is associated with glioma pathological malignant progression and immune phenotype in the Qilu dataset. **A **GSVA enrichment analysis showing the activation status of biological pathways in the HSPA7-high and HSPA7-low groups. Heatmap was used to visualize these biological processes. Yellow represents activated pathways, black represents moderately activated pathways, and blue represents inhibited pathways. **B **GSEA showing the classical cancer-promoting pathways and immune-related pathways were signficantly enriched in the high TMSB10 expression glioma samples in Qilu dataset. **C **Abundances of immune/stromal score, tumorpurity and 25 immune cell types in TMSB10 high glioma samples versus low samples in Qilu dataset. The upper and lower ends of the boxes indicate the interquartile range of the values. The lines in the boxes represent the median values, and black dots show outliers. The significance of differences between the three clusters were determined by the Mann-Whitney U test. The statistical significance is indicated as follows: ns>0.05; *P < 0.05; **P < 0.01; ***P < 0.001. Bar graph of (**D**) enriched terms, colored by p-values, and (**E**) summary of enrichmentanalysis in TRRUST across genes positively correlated with TMSB10 in Qilu glioma dataset. **Figure S7.** TMSB10 promotes the proliferation, migration and invasion of glioma cells in vitro. **A **QRT-PCR assays showing the relative expression of TMSB10 in GBM cells and GSCs transfected with knocking down TMSB10 (sh-WEE2-AS1) or corresponding negative contorl (sh-NC). **B **Colony-forming assays showing the proliferation ability of GBM cells transfected with sh-NC or sh-TMSB10. **C **EDU assays showing the proliferation ability of GBM cells transfected with sh-NC or sh-TMSB10, scale bar, 50μm. Quantification histogram represented cell population. Data represented mean ± SD from at least three independent experiments. **D **QRT-PCR assays showing the relative expression of TMSB10 in GBM cells and GSCs overexpressing WEE2-AS1 (ov-WEE2-AS1) or corresponding negative control (ov-NC). **E **Wound Healing assays showing the migration ability of GBM cells transfected with sh-NC or sh-TMSB10. (**F-G) **Western blot assays showing the protein expression of MMP-9, N-cadherin, bcl2, bax, p21, CDK4 and cyclin D1 expression in GBM cells and mice tumor samples transfected with sh-NC or sh-TMSB10. Amounts of protein determined by densitometry of protein bands from three experiments. GAPDH was the loading control. Data represented mean ± SD from at least three independent experiments. The statistical significance is shown as: *P < 0.05; **P < 0.01; ***P < 0.001; ****P < 0.0001. **Figure S8**. TMSB10 regulates the cell cycle and apoptosis of glioma cells in vitro.** (A, B)** Cell cycle analysis for U87MG and U251 cells transfected with sh-NC or sh-TMSB10. The percentage of cells arrested in the G1/S phase is analyzed in a histogram (right panels). **C **Representative flow cytometry plots of cell apoptosis and quantitative analysis are shown. **D **Western blot assays showing the protein expression of TMSB10 expression in GBM cells. **E-H **Western blot assays showing the protein expression of CD44, YAP1, LOX, as well as phosphorylation levels of AKT and ERK1/2 in GSCs transfected with sh-NC or sh-TMSB10 and ov-NC or ov-TMSB10 as indicated. Amounts of protein determined by densitometry of protein bands from three experiments. β-actin was the loading control. **I **Western blot assays showing the protein expression of SPP1 in THP-1 differentiated macrophages treated with CM from GSCs transfected with sh-NC or sh-TMSB10 and ov-NC or ov-TMSB10 as indicated. Amounts of protein determined by densitometry of protein bands from three experiments. β-actin was the loading control. Data represented mean ± SD from at least three independent experiments. The statistical significance is shown as: *P < 0.05; **P < 0.01; ***P < 0.001; ****P < 0.0001. **Figure S9.** TMSB10 promotes GBM MES transformation and facilitates macrophage infiltration. **A **Correlation between TMSB10 and suppressive immunomodulators in three GBM datasets. The color indicates the correlation coefficient. **B **Correlation between TMSB10 and suppressive immunomodulators in Qilu dataset. Angle of sector indicates the correlation coefficient. **C **Correlation between TMSB10 and CD44 in three GBM datasets. GSEA showing the (**D**) MES signature and **(E)** CORDENONSI_YAP_CONSERVED_SIGNATURE in the high TMSB10 expression group in the Lee Y and Wang GBM cohorts.

**Additional file 2: Table S1.** Genes signaficantly correlated with TMSB10 in CGGA glioma dataset. **Table S2.** Joint analysis of the differentially expressed mRNAs and proteins between TMSB10 high GBM group versus low GBM group. **Table S3.** Differentially phosphorylated proteins in TMSB10 high expression GBM samples versus low expression. **Table S4.** Joint analysis of the differentially expressed protein and protein phosphorylation between TMSB10 high GBM group versus low GBM group. **Table S5.** Differentially acylatedd proteins in TMSB10 high expression GBM samples versus low expression. **Table S6.** Joint analysis of the differentially expressed protein and protein acylation between TMSB10 high GBM group versus low GBM group. **Table S7.** Genes signaficantly correlated with TMSB10 in Qilu dataset. **Table S8.** Drugs significantly associated with TMSB10. **Table S9.** Sequences for siRNAs, shRNAs and primers for qRT-PCR.

## Data Availability

All data used in this work can be acquired from the TCGA database (http://cancergenome.nih.gov/), CGGA database (http://www.cgga.org.cn/), and the circRNA sequencing and mRNA sequencing data of our local samples have been deposited in the Genome Sequence Archive (GSA) under accession number CRA002339, and data were released when the paper was published. The processed data are available from the corresponding author upon reasonable request.

## Abbreviations

TMSs: Thymosin family genes
TMSB10: Thymosin β10
TCGA: The Cancer Genome Atlas
ssGSEA: Single-sample gene set enrichment analysis
GSVA: Gene set variation analysis
TME: Tumor microenvironment
PTMA: Prothymosin alpha
PTMS: Parathymosin
ICIs: Immune checkpoint inhibitors
TAMs: Tumor-associated macrophages
CNA: Copy number alteration
HRs: Hazard ratios
CCLE: Cancer Cell Line Encyclopedia
GTEx: Genotype-Tissue Expression
LGG: Lower grade glioma
GBM: Glioblastoma
CAFs: Cancer-associated fibroblasts
CGGA: Chinese Glioma Genome Atlas
ROC: Receiver operating characteristic
GSEA: Gene set enrichment analysis
KEGG: Kyoto Encyclopedia of Genes and Genomes
GO: Gene ontology
GSCs: Glioma stem cells
PN: Proneural
CL: Classical
MES: Mesenchymal
BMDMs: Bone marrow-derived macrophages
MGs: Microglial cells
GBO: Glioblastoma organoid
