# Supplementary material for: Pancancer landscape analysis of the thymosin family identified TMSB10 as a potential prognostic biomarker and immunotherapy target in glioma
Source: Cancer Cell Int. 2022 Sep 26;22:294. doi: 10.1186/s12935-022-02698-5 (PMC9513945; doi:10.1186/s12935-022-02698-5)
Supplement: Supplementary file 1 — Additional file 1: Figure S1. The expression of TMSs in cancer cell lines in CCLE dataset. The expression of (A) TMSB10, (B)TMSB4X, (C) TMSB4Y, (D) TMSB15A, (E) TMSB15B, (F) PTMA and (G) PTMS in cancer cell lines in CCLE dataset. Figure S2. The expression of TMSs in normal tissues in GETx dataset. The expression of (A) TMSB10, (B)TMSB4X, (C) TMSB4Y, (D) TMSB15A, (E) TMSB15B, (F) PTMA and (G) PTMS in normal tissues in GETx dataset. Figure S3. The expression of TMSs between GTEx normal tissues and paired tumor tissues. The expression of (A)TMSB4X, (B) TMSB4Y, (C) TMSB15A, (D) TMSB15B, (E) PTMA and (F) PTMS between GTEx normal tissues and tumor tissues. The asterisks indicated a statistically significant p-value calculated using Mann-Whitney U test. The statistical significance is indicated as follows: ns>0.05; *P < 0.05; **P < 0.01; ***P < 0.001. Figure S4. Biological pathways and immunological characteristics of TMSB10 in glioma. A Kaplan–Meier curves for the OS of GBM patients with high TMSB10 expression and low TMSB10 expression in three GBM datasets; the log-rank test was used to calculate the p value. B Correlation between TMSB10 and the infiltration of 25 tumor-associated TME cells calculated with the ssGSEA algorithm. The color indicates the correlation coefficient. C Spearman correlation analysis of TMSB10 and classical signaling pathways in three glioma cohorts. Red indicates positive correlations, and the darkness of color is proportional to the correlation coefficient. The size of the circle represents the statistical P value, with larger circles representing greater statistical significance. Bar graph of (D) enriched terms, colored by p-values, and (E) summary of enrichmentanalysis in TRRUST across genes positively correlated with TMSB10 in CGGA glioma dataset. Figure S5. Multiomics regulatory profile of TMSB10 in glioma. A KEGG enrichment analysis of genes with significantly upregulated at both mRNA and protein levels in GBM samples with high TMSB10 expre [file 12935_2022_2698_MOESM1_ESM.docx]

**Figures**


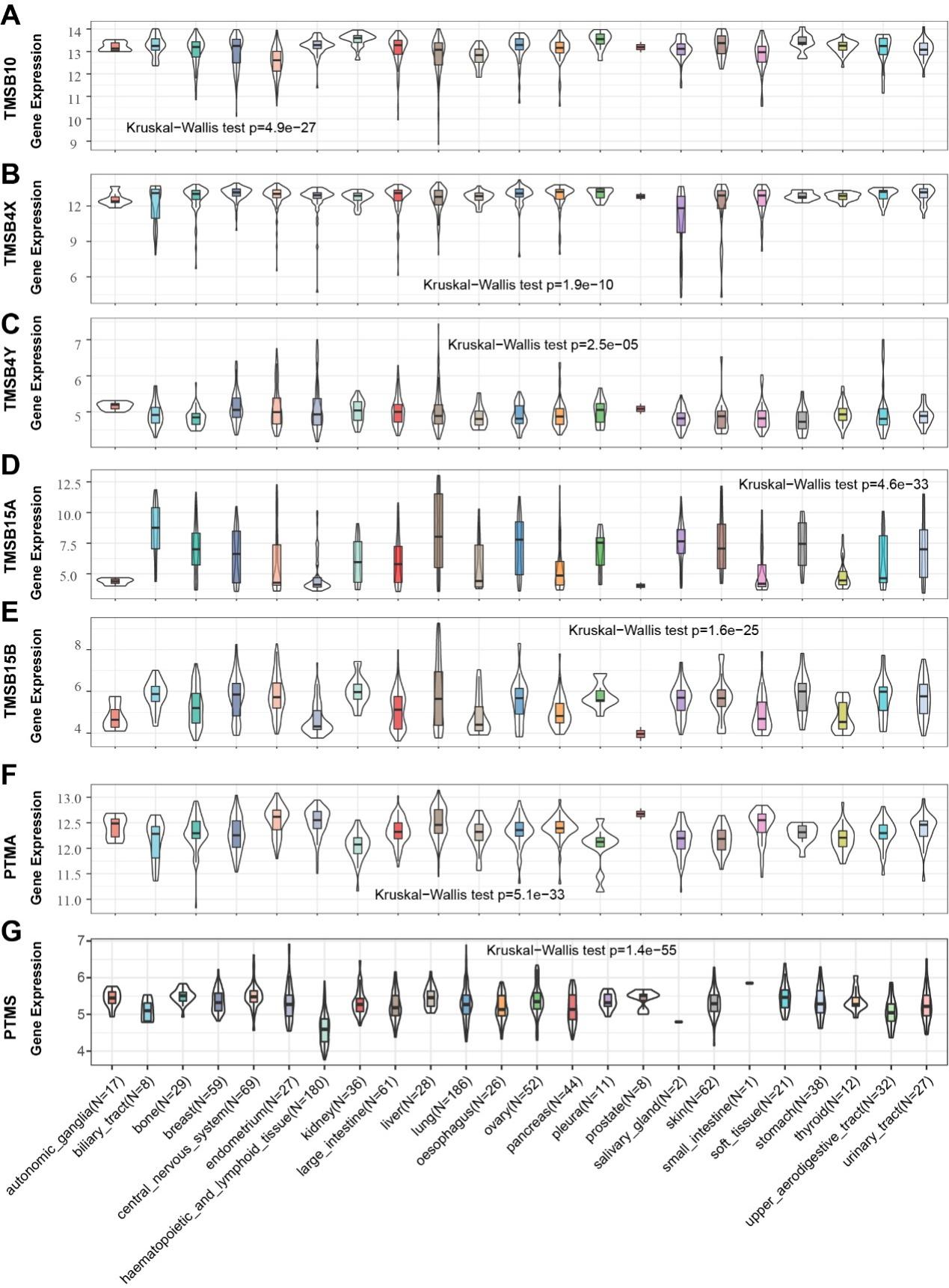


**Figure S1. The expression of TMSs in cancer cell lines in CCLE dataset.** The expression of **(A)** TMSB10, **(B)**TMSB4X, **(C)** TMSB4Y, **(D)** TMSB15A, **(E)** TMSB15B, **(F)** PTMA and **(G)** PTMS in cancer cell lines in CCLE dataset.


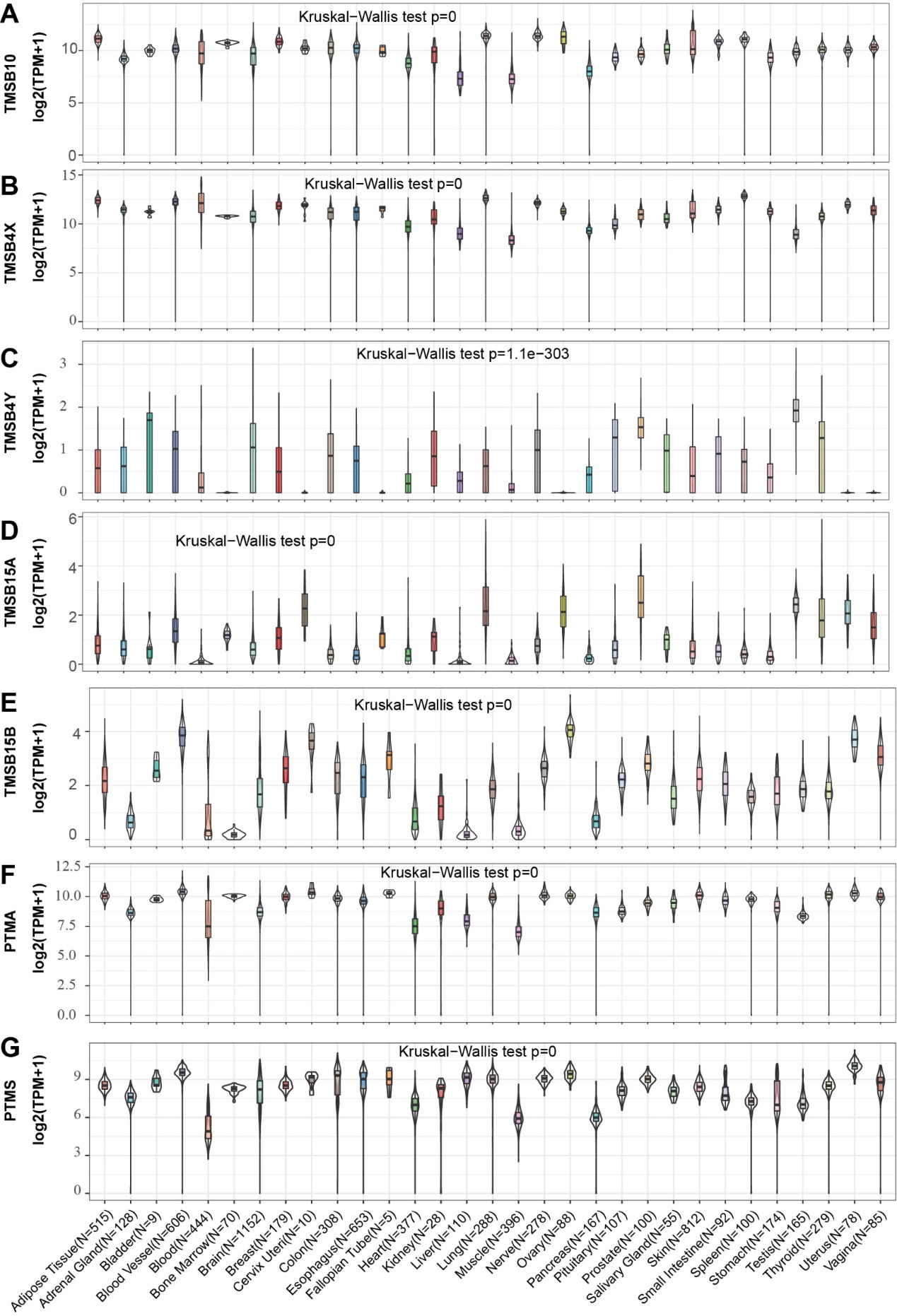


**Figure S2. The expression of TMSs in normal tissues in GETx dataset.** The expression of **(A)** TMSB10, **(B)**TMSB4X, **(C)** TMSB4Y, **(D)** TMSB15A, **(E)** TMSB15B, **(F)** PTMA and **(G)** PTMS in normal tissues in GETx dataset.


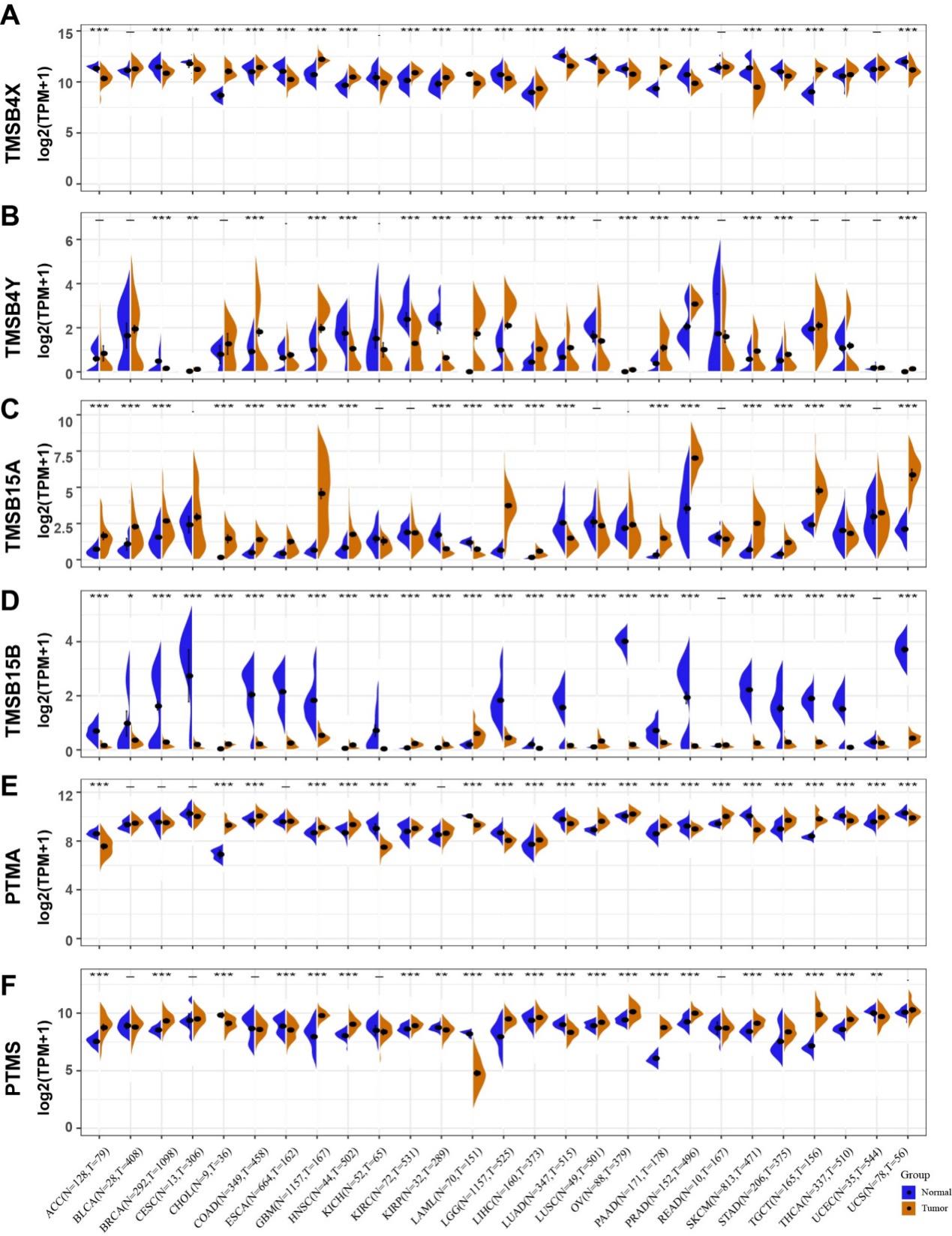


**Figure S3**. The expression of TMSs between GTEx normal tissues and paired tumor tissues. The expression of **(A)**TMSB4X, **(B)** TMSB4Y, **(C)** TMSB15A, **(D)** TMSB15B, **(E)** PTMA and **(F)** PTMS between GTEx normal tissues and tumor tissues. The asterisks indicated a statistically significant p-value calculated using Mann-Whitney U test. The statistical significance is indicated as follows: ns>0.05; *P < 0.05; **P < 0.01; ***P < 0.001.


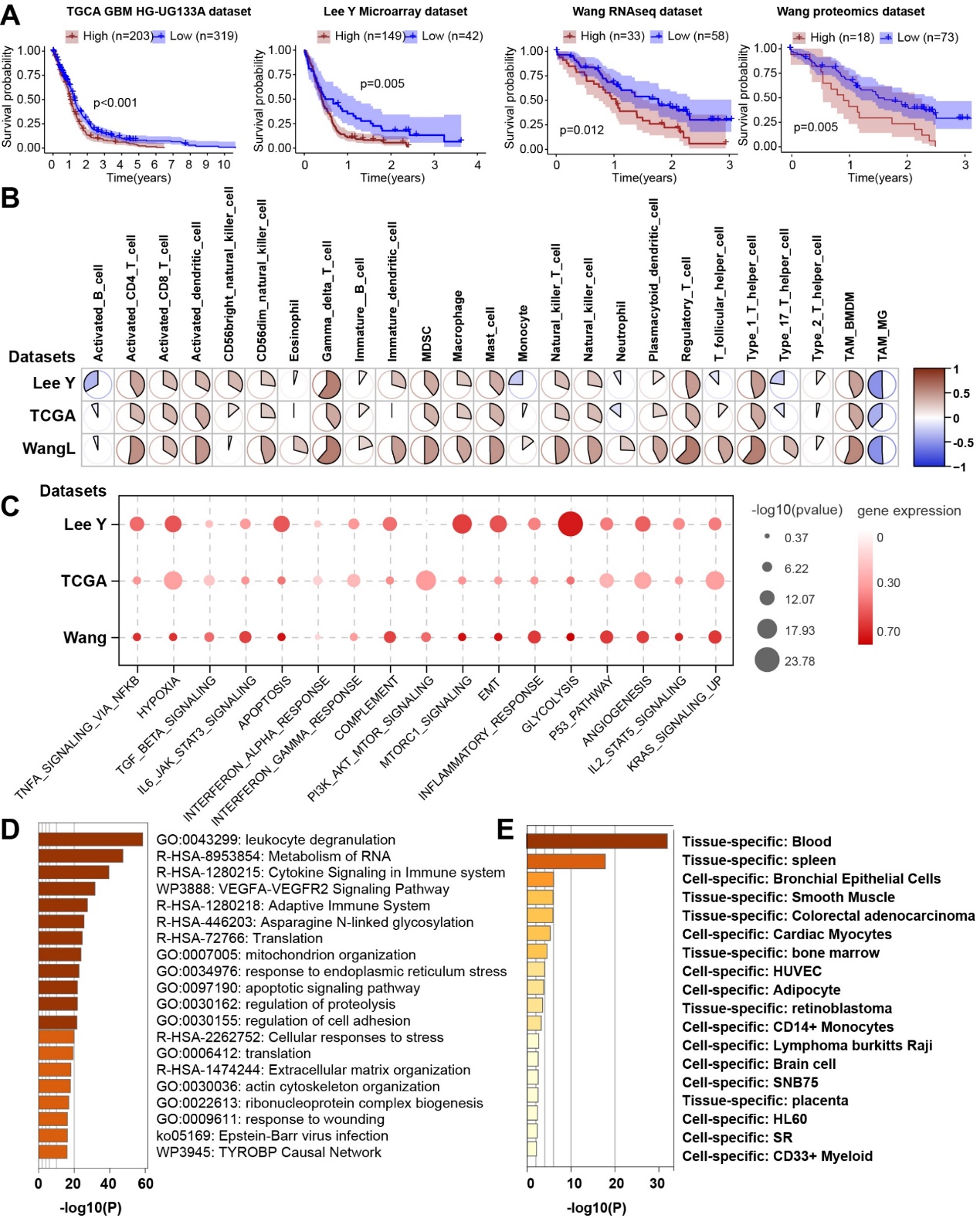


**Figure S4. Biological pathways and immunological characteristics of TMSB10 in glioma. A** Kaplan–Meier curves for the OS of GBM patients with high TMSB10 expression and low TMSB10 expression in three GBM datasets; the log-rank test was used to calculate the p value. **B** Correlation between TMSB10 and the infiltration of 25 tumor-associated TME cells calculated with the ssGSEA algorithm. The color indicates the correlation coefficient. **C** Spearman correlation analysis of TMSB10 and classical signaling pathways in three glioma cohorts. Red indicates positive correlations, and the darkness of color is proportional to the correlation coefficient. The size of the circle represents the statistical P value, with larger circles representing greater statistical significance. Bar graph of (**D**) enriched terms, colored by p-values, and (**E**) summary of enrichmentanalysis in TRRUST across genes positively correlated with TMSB10 in CGGA glioma dataset.


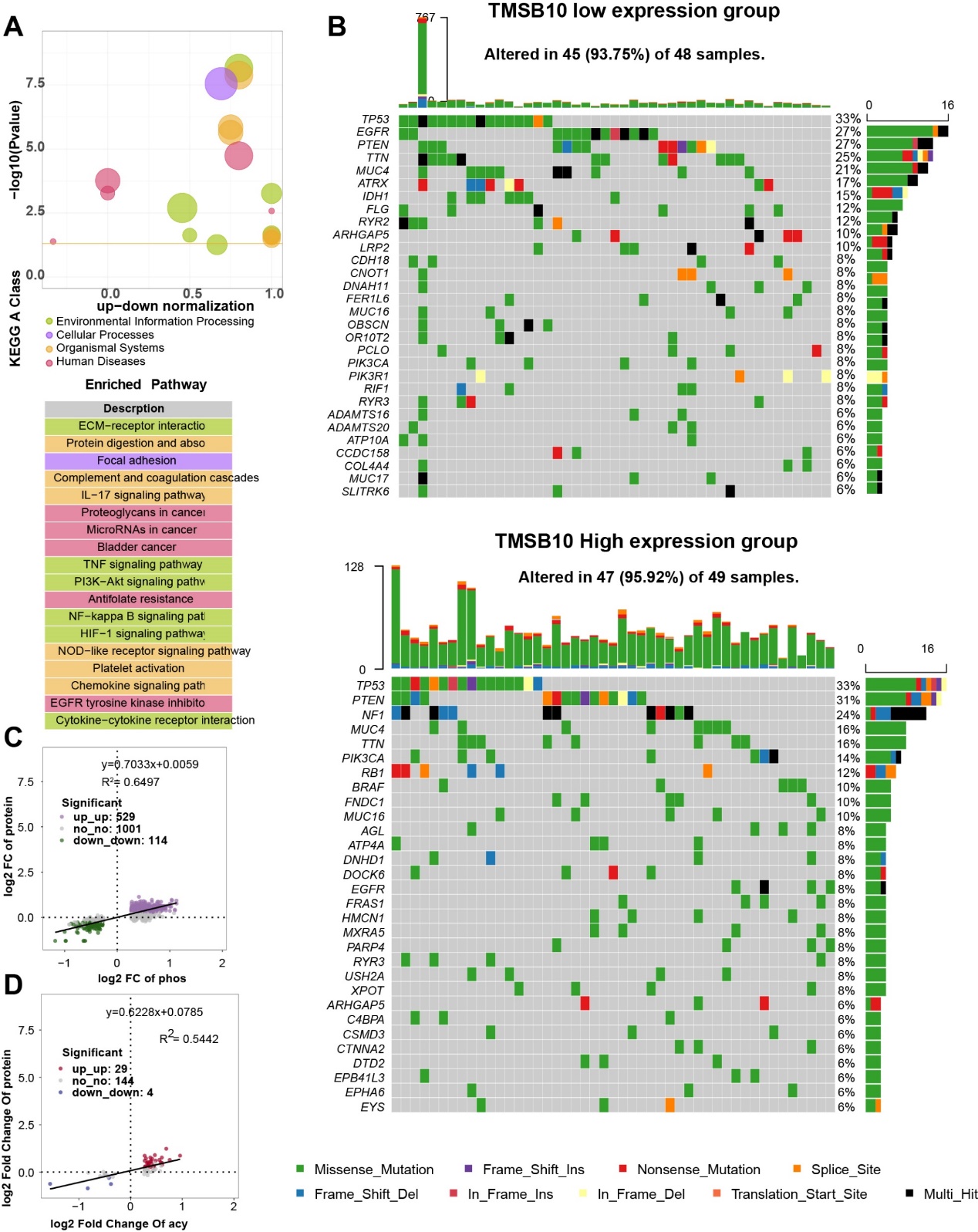


**Figure S5. Multiomics regulatory profile of TMSB10 in glioma. A** KEGG enrichment analysis of genes with significantly upregulated at both mRNA and protein levels in GBM samples with high TMSB10 expression. **B** Waterfall plot of the tumor somatic mutation landscape in the (upper) low-TMSB10 and (lower) high-TMSB10 samples in the wang GBM dataset. Dot plot of the log2FC (protein expression) versus the (**C**) log2FC (protein phosphorylation expression), and (**D**) log2FC (protein acylation expression), showing a positive correlation between the overall protein phosphorylation/acylation level and protein expression and the distribution of genes with significant changes in both the protein expression (|FC| > 1.2, P < 0.05) and corresponding protein phosphorylation/acylation expression (|FC| > 1.2, P < 0.05) in the high TMSB10 expression group compared with the low TMSB10 expression group.


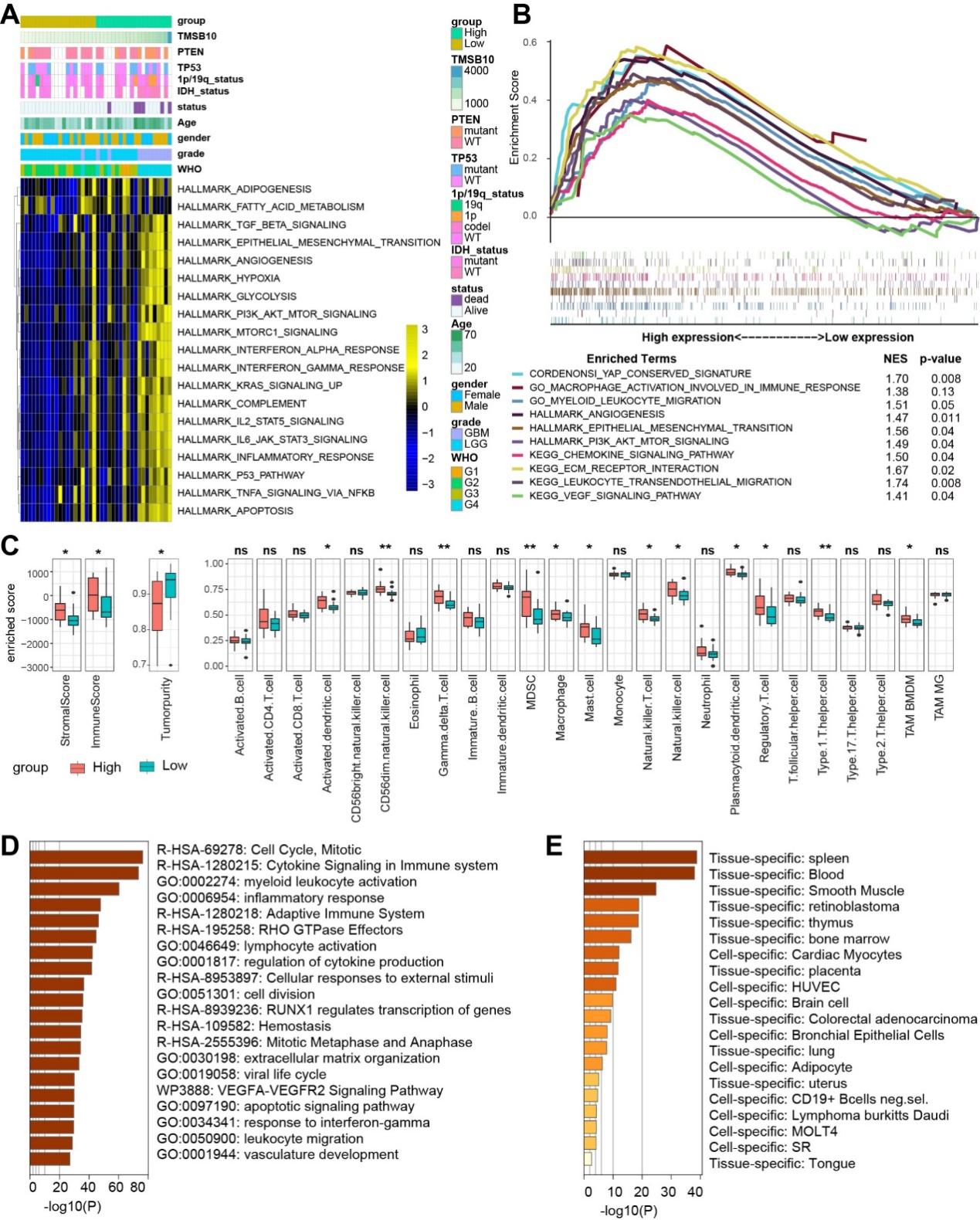


**Figure S6. TMSB10 expression is associated with glioma pathological malignant progression and immune phenotype in the Qilu dataset. A** GSVA enrichment analysis showing the activation status of biological pathways in the HSPA7-high and HSPA7-low groups. Heatmap was used to visualize these biological processes. Yellow represents activated pathways, black represents moderately activated pathways, and blue represents inhibited pathways. **B** GSEA showing the classical cancer-promoting pathways and immune-related pathways were signficantly enriched in the high TMSB10 expression glioma samples in Qilu dataset. **C** Abundances of immune/stromal score, tumorpurity and 25 immune cell types in TMSB10 high glioma samples versus low samples in Qilu dataset. The upper and lower ends of the boxes indicate the interquartile range of the values. The lines in the boxes represent the median values, and black dots show outliers. The significance of differences between the three clusters were determined by the Mann-Whitney U test. The statistical significance is indicated as follows: ns>0.05; *P < 0.05; **P < 0.01; ***P < 0.001. Bar graph of (**D**) enriched terms, colored by p-values, and (**E**) summary of enrichmentanalysis in TRRUST across genes positively correlated with TMSB10 in Qilu glioma dataset.


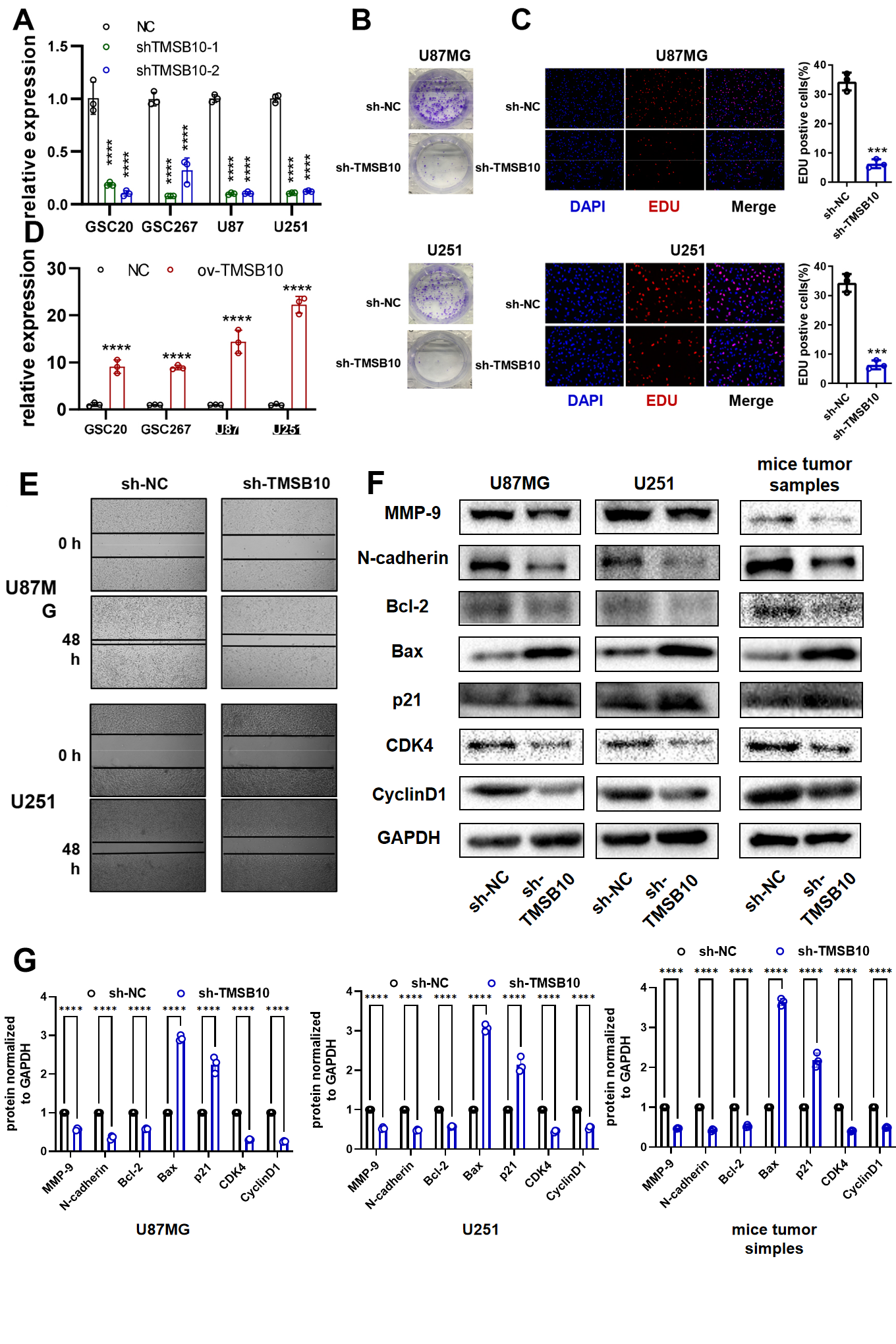


**Figure S7. TMSB10 promotes the proliferation, migration and invasion of glioma cells in vitro. A** QRT-PCR assays showing the relative expression of TMSB10 in GBM cells and GSCs transfected with knocking down TMSB10 (sh-WEE2-AS1) or corresponding negative contorl (sh-NC). **B** Colony-forming assays showing the proliferation ability of GBM cells transfected with sh-NC or sh-TMSB10. **C** EDU assays showing the proliferation ability of GBM cells transfected with sh-NC or sh-TMSB10, scale bar, 50μm. Quantification histogram represented cell population. Data represented mean ± SD from at least three independent experiments. **D** QRT-PCR assays showing the relative expression of TMSB10 in GBM cells and GSCs overexpressing WEE2-AS1 (ov-WEE2-AS1) or corresponding negative control (ov-NC). **E** Wound Healing assays showing the migration ability of GBM cells transfected with sh-NC or sh-TMSB10. (**F-G)** Western blot assays showing the protein expression of MMP-9, N-cadherin, bcl2, bax, p21, CDK4 and cyclin D1 expression in GBM cells and mice tumor samples transfected with sh-NC or sh-TMSB10. Amounts of protein determined by densitometry of protein bands from three experiments. GAPDH was the loading control. Data represented mean ± SD from at least three independent experiments. The statistical significance is shown as: *P < 0.05; **P < 0.01; ***P < 0.001; ****P < 0.0001.


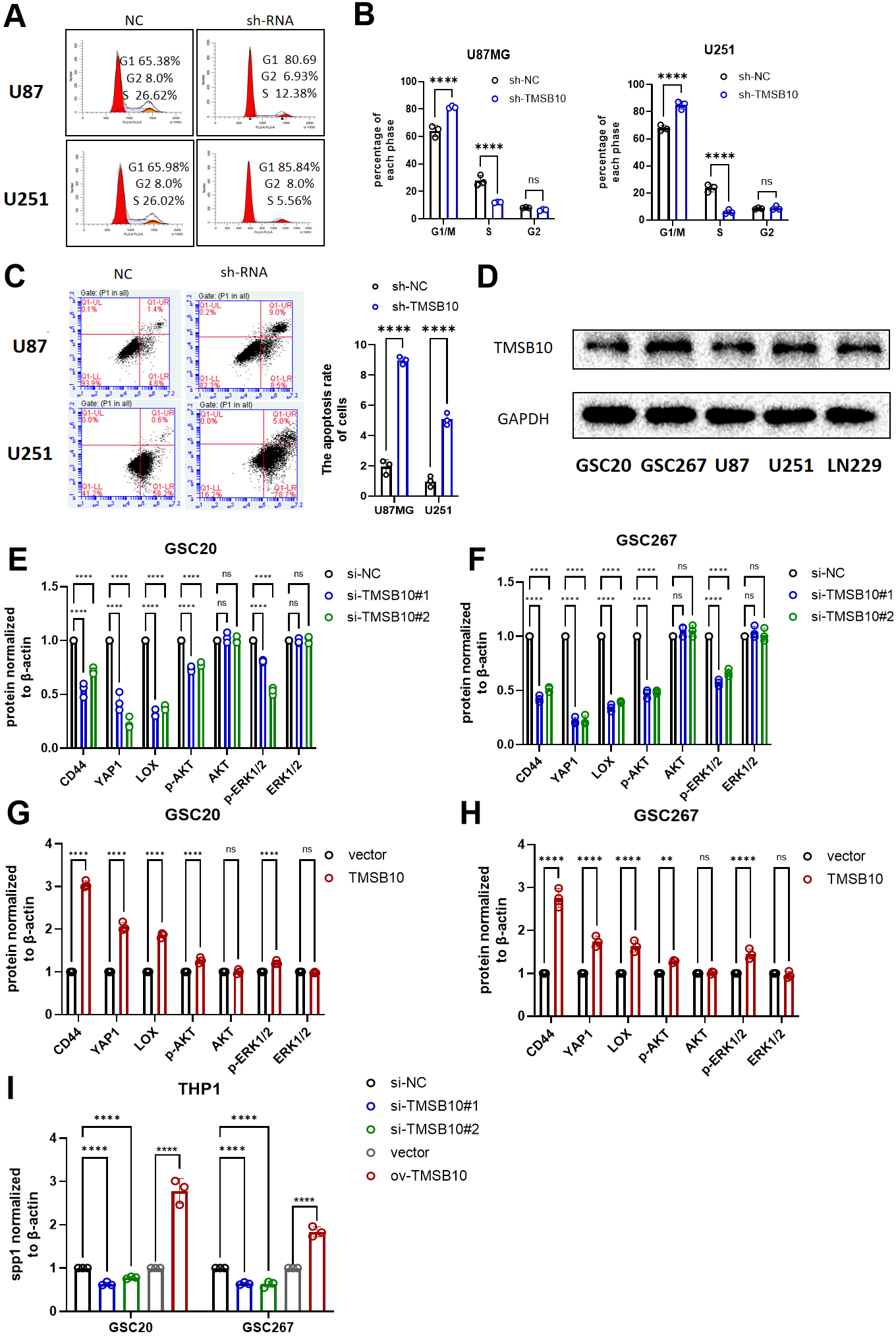


**Figure S8. TMSB10 regulates the cell cycle and apoptosis of glioma cells in vitro. (A, B)** Cell cycle analysis for U87MG and U251 cells transfected with sh-NC or sh-TMSB10. The percentage of cells arrested in the G1/S phase is analyzed in a histogram (right panels). **C** Representative flow cytometry plots of cell apoptosis and quantitative analysis are shown. **D** Western blot assays showing the protein expression of TMSB10 expression in GBM cells. **E-H** Western blot assays showing the protein expression of CD44, YAP1, LOX, as well as phosphorylation levels of AKT and ERK1/2 in GSCs transfected with sh-NC or sh-TMSB10 and ov-NC or ov-TMSB10 as indicated. Amounts of protein determined by densitometry of protein bands from three experiments. β-actin was the loading control. **I** Western blot assays showing the protein expression of SPP1 in THP-1 differentiated macrophages treated with CM from GSCs transfected with sh-NC or sh-TMSB10 and ov-NC or ov-TMSB10 as indicated. Amounts of protein determined by densitometry of protein bands from three experiments. β-actin was the loading control. Data represented mean ± SD from at least three independent experiments. The statistical significance is shown as: *P < 0.05; **P < 0.01; ***P < 0.001; ****P < 0.0001.


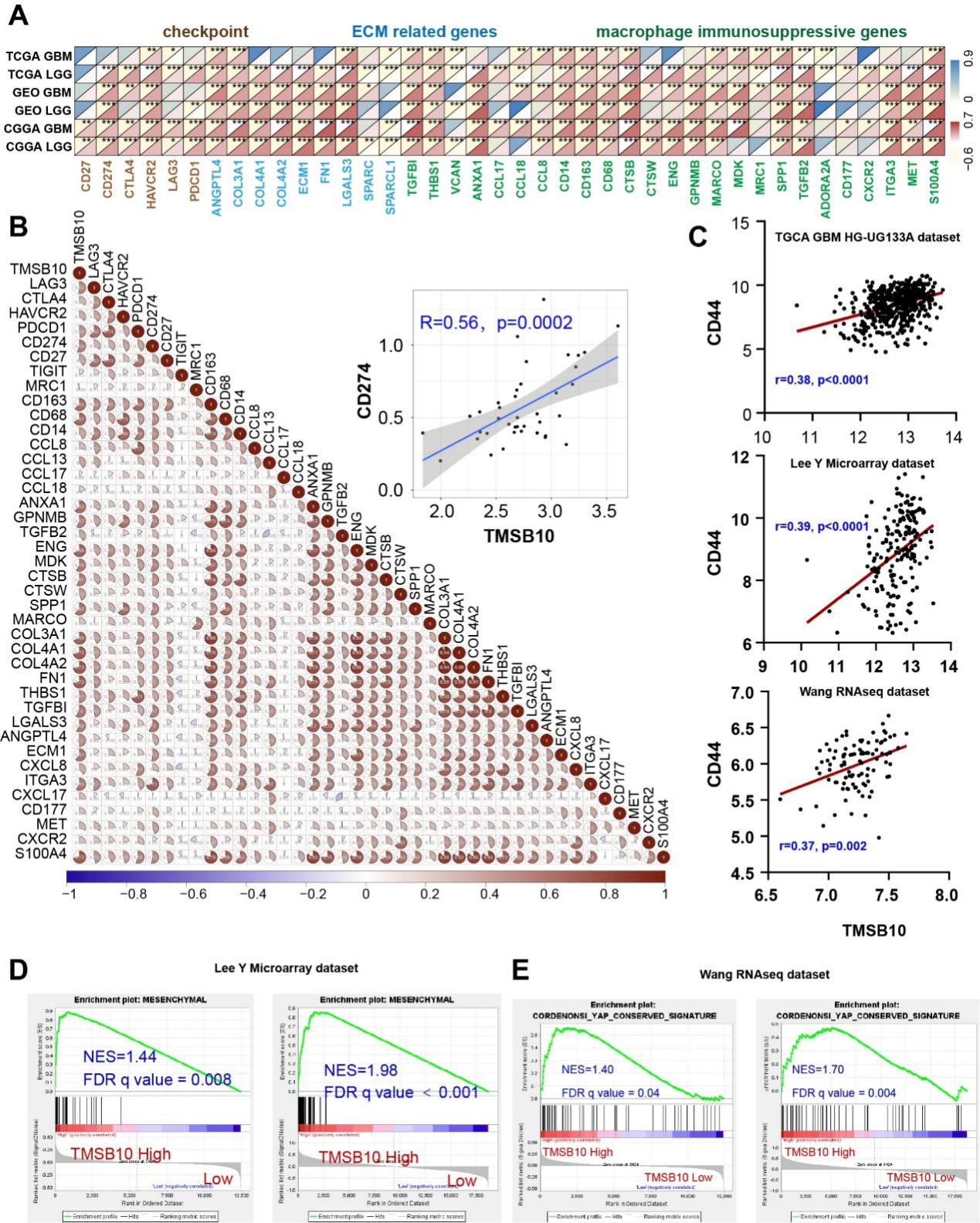


**Figure S9. TMSB10 promotes GBM MES transformation and facilitates macrophage infiltration. A** Correlation between TMSB10 and suppressive immunomodulators in three GBM datasets. The color indicates the correlation coefficient. **B** Correlation between TMSB10 and suppressive immunomodulators in Qilu dataset. Angle of sector indicates the correlation coefficient. **C** Correlation between TMSB10 and CD44 in three GBM datasets. GSEA showing the (**D**) MES signature and **(E)** CORDENONSI_YAP_CONSERVED_SIGNATURE in the high TMSB10 expression group in the Lee Y and Wang GBM cohorts.

**Supplementary Methods**

# Collection and preprocessing of publicly available pan-cancer expression datasets

The RNA-seq data (RPKM format) and corresponding clinical information for patients with 33 types of cancer (ACC: adrenocortical carcinoma; BLCA: bladder urothelial carcinoma; BRCA: breast invasive carcinoma; CESC: cervical squamous cell carcinoma; CHOL: cholangiocarcinoma; COAD: colon adenocarcinoma; DLBC: lymphoid neoplasm diffuse large B cell lymphoma; ESCA: esophageal carcinoma; GBM: Glioblastoma multiforme; LGG: brain lower grade glioma; HNSC: head and neck squamous cell carcinoma; KICH: kidney chromophobe; KIRC: kidney renal clear cell carcinoma; KIRP: kidney renal papillary cell carcinoma; LAML: acute myeloid leukemia; LIHC: liver hepatocellular carcinoma; LUAD: lung adenocarcinoma; LUSC: lung squamous cell carcinoma; MESO: mesothelioma; OV: ovarian serous cystadenocarcinoma; PAAD: pancreatic adenocarcinoma; PCPG: pheochromocytoma and paraganglioma; PRAD: prostate adenocarcinoma; READ: rectum adenocarcinoma; SARC: sarcoma; SKCM: skin cutaneous melanoma; STAD: stomach adenocarcinoma; TGCT: testicular germ cell tumor; THCA: thyroid carcinoma; THYM: Thymoma; UCEC: uterine corpus endometrial carcinoma; UCS: uterine carcinosarcoma; and UVM: uveal melanoma) were obtained from TCGA. All expression data were then transformed into TPM format.

The basal expressions of TMSs family genes were investigated based on GTEx datasets. And expression data of TMSs in cancer cell lines were downloaded from Cancer Cell Line Encyclopedia (CCLE) project.

# Somatic mutation and copy number alteration analysis

TCGA pan-cancer somatic mutation data was downloaded from Genomic Data Commons. We performed multiple filtering steps to eliminate artifacts and reduce false-positive rates as previous described [35]. Briefly, in the file table, the values in the FILTER column are required to be one of wga, native_wga_mix or PASS, and only non-silent mutations retained (the values in Variant_Classification column should be one of Frame_Shift_Del, Frame_Shift_Ins, In_Frame_Del, In_Frame_Ins, Missense_Mutation, Nonsense_Mutation, Nonstop_Mutation, Splice_Site, and Translation_Start_Site). TCGA thresholded SCNA scores for 9125 patient samples from Genome Data Commons (ISAR-corrected GISTIC2.0 all_thresholded.by_genes file). Values equal to 2 and −2 were considered to be amplification and deep deletion, respectively, as previous described. OncoPrint plot of mutation and SCNA was generated by using ComplexHeatmap R package.

# Collection and preprocessing of publicly available other glioma expression datasets

The CGGA RNA-seq datasets (325 samples) and their clinical information were downloaded from the CGGA database (<http://www.cgga.org.cn/>), the Gravendeel microarray dataset and clinical information were downloaded from the GlioVis database (<http://gliovis.bioinfo.cnio.es/>), the Wang RNA-seq dataset (FPKM format) and clinical information were extracted from the supplemental data of the article, and the missing data were obtained with the K-nearest neighbor (KNN) method. For ICB data, we downloaded the raw data from SRA PRJNA482620 and then processed them into TPM format, and clinical information was obtained from the supplemental data of the article.

# Biological pathway analysis

To explore the biological behaviours among distinct samples, we used some gene sets of HALLMARK[38] from the MSigDB database to estimate pathway enrichment scores for each sample for GSVA enrichment analysis using the “GSVA” R package. GSEA was also used to estimate the enrichment of various biological processes in each sample. And genes positively correlated with TMSB10 were analysed using the web tool “Metascape” for pathway and process enrichment analysis (https://metascape.org/gp/index.html).

# Cox regression analysis

The univariate Cox regression analyses were performed based on the expression of TMSs in cancers. The multivariate analyses were performed based on the TMSB10 gene expression and patient clinical information, such as glioma grade, age and IDH status.

# Differential expression analysis and functional analysis

We classified patients into two groups based on the median cut-off expression of circNEIL3 in our local dataset or IGF2BP3 in the TCGA glioma dataset. Then, differential expression analysis was analysed using the R package “DEseq2”. Furthermore, upregulated genes were analysed using the web tool “Metascape” for pathway and process enrichment analysis (<https://metascape.org/gp/index.html>).

# Integrated transcriptome and proteome analysis

The proteomic, phosphoproteomic, acetylomic, lipidomic and metabolomic data in the Wang dataset were acquired from the supplemental data of the paper.

Transcriptome and proteomic data in the Wang dataset were acquired from the supplemental data of the paper. The differentially expressed mRNAs and proteins between the high and low TMSB10 expression groups were screened by using the “limma” package in R language. For transcriptome data, a Padj<0.05 and |FC|> 2 were considered the cutoff values for determining DEGs. For proteomic data, a Padj <0.05 and |FC|>1.2 were considered the cutoff values for determining differentially expressed proteins. A nine-quadrant diagram showing genes with transcriptional and translational expression differences was constructed.

# Integrated proteome and phosphoproteomics/acetylomics analysis

The differential phosphosite/acetylomics abundances between the high and low TMSB10 groups were also analyzed by using the “limma” package in R language. Padj <0.05 and |FC|>1.2 were used as the cutoff values for determining differential phosphosite/acetylomics values, and the proteins with significantly upregulated or downregulated phosphosite/acetylomic values were considered as differential phosphoproteins. The overall trends of the relationships between protein phosphorylation/acetylation levels and their corresponding protein expression and the distribution of differential phosphorylation/acetylation sites and their corresponding protein expression differences were visualized by two histologies distributed in two dimensions (i.e., the horizontal X-axis and vertical Y-axis).

# Tumor mutation distribution analysis

The corresponding mutation data of patients in the TCGA LGG and GBM cohort were downloaded from the TCGA data portal (<https://www.cancer.gov/tcga/>). The corresponding mutation data of patients in the Wang cohort were obtained from the supplementary data of the study. The waterfall function of the “maftools” package was used to show the difference in mutation distribution between patients in the high and low TMSB10 groups.

# Western Blot

Whole-cell protein was extracted from glioma cells, GSCs and monocyte cells. The protein was transferred to a nitrocellulose membrane and incubated with specific antibodies. The following primary antibodies were used: CD44 (Cell Signaling Technology, 3570), LOX (Abcam, ab174316), and YKL40 (Cell Signaling Technology, 47066), SPP1 (Abcam, ab8448).

# RNA extraction

Total RNA was extracted using RNA-Quick Purification Kit (ESscience Biotech, China) according to the manufacturer’s instructions. cDNA was synthesized using ReverTra Ace qPCR RT Master Mix (Toyobo, Japan).

# Transwell and wound healing assays

For transwell assay, 2×104 cells were seeded in the upper chamber of a transwell insert (Corning) with or without Matrigel (BD bioscience). After incubation for 24h, cells that passed through the membrane were counted. For wound healing assay, confluent monolayers of cells were planted into 6-well plates and scratched with a 200 μl plastic pipette tip. The migration of cells was photographed at 0h and 48h time points.

# Cell proliferation assays

For CCK-8 assay, 5×103 cells were seeded into the 96-well plates. CCK-8 solution was added into the plates with a 1:10 ratio. After incubation at 37°C for 2h, the absorbance was measured at a wave-length of 450nm. For colony formation assay, the glioma cells were seeded into 6-well plates after transfection. After 14 days, samples were fixed with paraformaldehyde and stained with crystal violet for counting. For EdU assay, cells were planted into 48-well plates and added with EdU. After 4 hours, the cells were fixed by paraformaldehyde and stained by Apollo®567 and Hoechst (C10310-1, RIOBIO).

# Immunofluorescence

The slides were incubated with the following primary antibodies: CD68 (Abcam, ab213363), SPP1 (Abcam, ab8448), TMSB10 (Santa cruz, sc-514309). After the incubation, the samples were treated with the matching fluorescent secondary antibodies (1:500 dilution, Thermo Fisher). LeicaSP8 confocal microscope (Leica Microsystems, Wetzlar, Germany) was used to capture the images.

# Flow cytometry analysis

Anti-CD163-PE (BD Biosciences, USA) was used to stain macrophage for 30 min. Then cells were washed for 3 times with PBS and suspended in 300ul PBS. For the analysis of cell cycle, cells were fixed with 75% ethanol overnight. Next, the cells was incubated with 300μl of propidium iodide containing RNase for 15 min. For the analysis of apoptosis, FITC-Annexin V apoptosis detection kit (BD Biosciences, USA) was used to detect the percentage of apoptosis, according to the manufacturer’s protocol. BD Accuri C6 flow cytometer (BD Biosciences) and FlowJo 7.6.1 (Leonard Herzenberg) was used for flow cytometry analysis.

# Correlation analysis of the TMSB10 and drug sensitivity

Drug sensitivity data (Zscore values) and gene expression data were downloaded from the CellMiner database (https://discover.nci.nih.gov/cellminer/), and the drugs were screened. Any data that were not FDA approved and were not from clinical trials were excluded. We then performed Pearson correlation analysis to calculate the correlation between drug sensitivity and the TMSB10, and a P value < 0.05 was considered significant.
